# Temporal enrichment of bacterial monoamine oxidase gene aboard the international space station and neuromodulator impacts of dopamine-driven ammonium formed by space isolate *Leifsonia virtsii* on *Caenorhabditis elegans*

**DOI:** 10.3389/fmicb.2026.1943403

**Published:** 2026-10-23

**Authors:** Naikap Mayuri, Honagodu Ravichandra Dhanyashree, Rajesh Padumane Shastry, Kasthuri Venkateswaran, Georgios Miliotis, Nitin Kumar Singh, Paul Stothard, Ananthapadmanabha Bhagwath Arun, Asif Hameed

**Affiliations:** 1 Division of Microbiology and Biotechnology, Yenepoya Research Centre, Yenepoya (Deemed to be University), Deralakatte, Mangalore, India; 2 Caenorhabditis elegans Laboratory Facility (CeLF), Yenepoya Research Centre, Yenepoya (Deemed to be University), Deralakatte, Mangalore, India; 3 Anaerobic & Translational Genomics Centre (ATGC), Yenepoya Research Centre, Yenepoya (Deemed to be University), Deralakatte, Mangalore, India; 4 NASA Jet Propulsion Laboratory (JPL), California Institute of Technology, Pasadena, CA, United States; 5 Department of Space Studies, University of North Dakota, Grand Forks, ND, United States; 6 Antimicrobial Resistance and Microbial Ecology Group, School of Medicine, University of Galway, Galway, Ireland; 7 Department of Industrial Relations, Division of Occupational Safety and Health, Oakland, CA, United States; 8 Department of Agricultural, Food and Nutritional Science, University of Alberta, Edmonton, AB, Canada; 9 Yenepoya International School, Al Khobar, Saudi Arabia

**Keywords:** actinomycetes, chemotaxis, dopamine, metagenomics, Microbacteriaceae

## Abstract

Dopamine regulates neurological functions, whereas its deficiency is linked with cognitive impairment and neurodegenerative diseases. The spatiotemporal distribution of dopamine-degrading microbes and the implications of dopamine catabolites on eukaryotes remain unexplored. Here, we demonstrate dopamine metabolism in *Leifsonia virtsii* F6_8S_P_1A^T^, an Actinomycetes isolated from the crew quarters aboard the International Space Station (ISS), and reveal dopamine catabolite-driven modulation of cognition and chemotaxis in *Caenorhabditis elegans*. *L. virtsii* shared a high 16S rRNA gene sequence similarity (>97.9%) and OrthoANI values (79.44–84.96%) with the *Leifsonia* species, isolated from terrestrial and aquatic habitats. A key gene encoding monoamine oxidase (MAO) involved in dopamine catabolism was found in 61.5% of validly established *Leifsonia* species, including *L. virtsii*, but not in ISS co-isolate *L. williamsii* F6_8S_P_1B^T^. Analysis of the ISS metagenome revealed temporal enrichment of MAO-associated signatures across Microbial Tracking studies (MT1 to MT3, 2015-2022), driven predominantly by candidate MAO homologs taxonomically associated with Actinomycetota in MT3, whereas strong F6-8S-P-1A-like MAO signals were restricted to two MT2 libraries. Dopamine input stimulated MAO gene expression in *L. virtsii* at early growth phase, and was found to complement cell division, biofilm establishment and extracellular NH_4_^+^ formation in *L. virtsii*. MAO expression decreased with increasing time/NH_4_^+^ concentration, whereas expression of marker genes representing DNA synthesis and transcription (*gyrB*), translation (*tuf*) and biofilm formation (*cslA*) exhibited concurrent upregulation. Dopamine-driven NH_4_^+^ formation further altered cognition and chemotaxis in *C. elegans*. Our analysis suggests that the dopamine metabolic pathway detected in *L. virtsii* is not an ISS-specific trait and rather represents a phenotype retrieved from Earth. The present study provides direct evidence of spatiotemporal flexibility and heterogeneous dopamine-metabolizing capacity among Actinomycetes. The findings further demonstrate that a dopamine catabolite can modulate chemosensory interactions in a eukaryotic model and highlight the succession and persistence of MAO-bearing taxa aboard ISS.

## 1 Introduction

Several microorganisms that travelled with humans and cargo shipments during spaceflight now persist in the International Space Station (ISS), even after intense cleaning and microbial monitoring procedures (Mora et al., 2016; Checinska Sielaff et al., 2019; Urbaniak et al., 2022). Microbes found in ISS may withstand harsh space environments such as microgravity, high radiation, elevated CO_2_ levels, and nutrient scarcity (Horneck et al., 2010; Knox et al., 2016). Microbial tracking data showed prevalence of bacterial genera *Staphylococcus, Pseudomonas, Bacillus*, *Acinetobacter*, *Arthrobacter, Leifsonia, Paenibacillus,* and *Sporosarcina,* the yeast genus *Rhodotorula,* and fungal genera *Penicillium, Aureobasidium*, and *Aspergillus* aboard ISS (Checinska Sielaff et al., 2019). New species of *Arthrobacter* (*n* = 1), *Leifsonia* (*n* = 2) and *Microbacterium* (*n* = 2), belonging to the phylum Actinomycetota, were reported recently from ISS (Hill et al., 2025; Karouia et al., 2025; Simpson et al., 2023). The members of Actinomycetota are mostly of soil origin, but are also found in water and air and are generally regarded as harmless, while a few produce secondary metabolites that play crucial roles in the eukaryotic system (Rui et al., 2025). Some representatives of Actinomycetota, including *Arthrobacter* (Li et al., 2021), *Leifsonia* (De Carvalho et al., 2021; Han et al., 2013) and *Microbacterium* (Laffineur et al., 2003; Amano et al., 2019) have shown clinical association. In line with this, two novel *Microbacterium* species characterized from ISS exhibit unique antimicrobial-resistant and virulent phenotypes and/or found to be closely related to clinical counterparts (Hill et al., 2025; Karouia et al., 2025). This necessitates a detailed investigation on Actinomycetota inhabiting ISS.

Dopamine (DA) is a monoamine neurotransmitter in the catecholamine family, which also includes norepinephrine and epinephrine (Pagana and Pagana, 2024). DA is a major neurotransmitter, implicated in conditions such as depression, and is regarded as a key regulator of cognitive functions, including decision-making, attention, memory, motivation and reward (Gonzalez-Arancibia et al., 2019). Previous studies have demonstrated microbial interaction with host DA pathways, although these observations have largely been limited to terrestrial gut or clinical microbiomes. Levodopa, a precursor to DA, can be metabolized by *Enterococcus faecalis*, reducing the bioavailability of DA in the gut, which could affect the outcomes of the treatment of Parkinson’s disease (Cheng et al., 2024). Similarly, *Bacteroides uniformis* and *Prevotella copri* have been shown to modulate DA transport activity in host systems, suggesting microbial regulation of dopaminergic signalling (Agusti et al., 2021; Hamamah et al., 2022). DA, norepinephrine and other catecholamines are known to stimulate growth, iron acquisition, virulence, and biofilm formation in pathogens such as *Salmonella enterica*, *Pseudomonas aeruginosa*, *Vibrio harveyi*, and *E. coli* (Yang et al., 2014; Dichtl et al., 2019; Xiang et al., 2024; Xu et al., 2026). The possible occurrence of microbes equipped with potential DA-metabolizing ability aboard the ISS and their implications in eukaryotic systems are not yet completely understood.

*Leifsonia virtsii* F6_8S_P_1A^T^ and *L. williamsii* F6_8S_P_1B^T^ (hereafter *L. virtsii* and *L. williamsii*, respectively) were isolated from crew quarters aboard the ISS in 2018 and characterized as two distinct species based on polyphasic taxonomy (Simpson et al., 2023). Genotypic analysis and phylogenomics indicated their close relationship with plant- and soil-associated *Leifsonia* species (Simpson et al., 2023). Our preliminary genome mining revealed the presence of MAO in *L. virtsii,* but not in *L. williamsii*, a co-isolate sharing the same niche. Analysis of *L. vitrsii* MAO at National Centre for Biotechnology Information (NCBI) and UniProt through the Basic Local Alignment Search Tool (BLAST) showed the occurrence of MAO homologs not only in some *Leifsonia,* but also in several other related members of the phylum Actinomycetota inhabiting Earth. However, neither MAO-bearing *Leifsonia* nor any other related species have been experimentally validated for actual DA-degrading phenotypes. Moreover, a comprehensive understanding of spatiotemporal flexibility of MAO-bearing species and impacts on eukaryotes is still lacking.

Here, we tested *L. vitrsii* using combined *in silico*, *in vitro* and *in vivo* experimental approaches for DA metabolism and its implication on a eukaryotic model system. *L. vitrsii* was chosen for in depth analysis mainly due to its ISS origin, where disturbance from confounding biotic factors remains low as compared to Earth-bound counterparts. In addition, temporal variations in MAO bearing taxa is much easier to trace due to the metagenomic microbial tracking missions carried out by NASA (MT1–3, 2015–2022) (Checinska Sielaff et al., 2019; Urbaniak et al., 2022). *L. williamsii* was tested in parallel as a representative MAO-lacking phenotype inhabiting ISS. At first, DA metabolic pathway (Mannisto and Kaakkola, 1999; Meiser et al., 2013) was reconstructed based on the genomic evidence to understand the intra-species distribution of DA-degrading genes in the genus *Leifsonia*. MAO-targeted metagenomics of ISS and MAO-based phylogenetic analysis were conducted to probe the congeneric and conspecific distribution of MAO across related taxa inhabiting manmade (ISS) and natural habitats (terrestrial and aquatic). Temporal variations in MAO-positive taxa were traced using the ISS indoor metagenome (PRJNA438545, PRJNA781277 and PRJNA1328788). DA metabolism was assessed directly in MAO-bearing *L. virtsii* using alamar blue and phenol red indicators, where *L. williamsii* served as an MAO-negative control. Planktonic cells, extracellular proteins, biofilm and NH_4_^+^-N was estimated as a function of DA input. The time course of the expression of genes involved in DA catabolism/NH_4_^+^ formation (*mao*), DNA synthesis and transcription (*gyrB*), translation (*tuf*) and biofilm formation (*cslA*) was probed. The *in silico* and *in vitro* findings were extrapolated to identify and validate a key DA catabolite that modulates cognition and chemotaxis in *C. elegans in vivo*.

## 2 Materials and methods

### 2.1 Strains and culture conditions

*L. virtsii* and *L. williamsii* were from the ISS (Simpson et al., 2023). The strains were cultured in Tryptic Soy Agar (TSA) (HiMedia, India) at 37 °C for 48 h, unless specified otherwise.

### 2.2 Pangenomics and phylogenetic analysis of MAO

16S rRNA gene sequence (OQ836671) of *L. virtsii* was used as a query in EzBiocloud (Yoon et al., 2017) (https://www.ezbiocloud.net/) to identify the closest related type strains of *Leifsonia* species in Orthologous Average Nucleotide Identity (OrthoANI) analysis (Lee et al., 2016). Subsequently, the LPSN (https://lpsn.dsmz.de/genus/leifsonia) database was mined to identify validly established *Leifsonia* (Table S1) and segregate reclassified species of *Leifsonia* (Table S2). The genomes of 13 authentic *Leifsonia* species (including *L. virtsii* and *L. williamsii*) were retrieved from NCBI database (Table S1), uploaded to RAST (Aziz et al., 2008) and screened for MAO, ALDH, and COMT homologs using corresponding reference proteins retrieved from UniProt. Comparative genomics was carried out through Proksee (Grant et al., 2023) using BLAST Tool 1.6.3 (Altschul et al., 1990) and BLAST+ 2.17.0. The genome of *L. virtsii* (GCA_030433675.1), annotated by NCBI PGAP 6.4, was compared with 12 *Leifsonia* genome assemblies (Table S1) using `blastn’, maintaining an E-value cut-off of 0.0001 by enabling low-complexity filtering. Bacterial code 11 was used as the genetic code. Each genome was placed on a separate ring. Strands were combined and hits were drawn by score. Nucleotide identity was displayed using the PuBu colour scale with boundaries at 0, 55, 60, 65, 70, 75, 80, 85, 90, 95, and 100%.

Closest-related homologs were retrieved from the NCBI database via BLASTp search and UniProt using *L. vitrsii* MAO as a query. Reference amino acid sequences corresponding to MAO were also retrieved from the in-house bacterial genome collections maintained at RAST for phylogenetic analysis. The MAO sequences were aligned using Clustal_X (Thompson et al., 1997). The evolutionary history was inferred by using the maximum likelihood method based on the JTT matrix-based model (Jones et al., 1992). The tree with the highest log-likelihood (−41423.8448) was taken into consideration. The percentage of trees in which the associated taxa clustered together is shown next to the branches. Initial tree(s) for the heuristic search were obtained by applying the neighbor-joining method to a matrix of pairwise distances estimated using a JTT model. The tree was drawn to scale, with branch lengths measured in the number of substitutions per site. The analysis involved 60 amino acid sequences. All ambiguous positions were removed for each sequence pair. There were a total of 808 positions in the final dataset. Evolutionary analyses were conducted in MEGA5 (Molecular Evolutionary Genetics Analysis, version 5.0) (Tamura et al., 2011). Tree topology was evaluated by using bootstrap resampling with 1,000 replicates (Felsenstein, 1985). The tree is drawn to scale, with branch lengths measured in the number of substitutions per site.

### 2.3 Screening ISS metagenome for MAO signatures and taxonomic assignments

The following eight ISS indoor sites were used consistently for three consecutive microbial tracking missions (MT3) of NASA. 1, Port panel next to the cupola; 2, Waste hygiene compartment; 3, Advanced resistive exercise device; 4, Dining table; 5, Overhead; 6, Permanent multipurpose module; 7, Lab 3 overhead; 8, Port crew quarters, bumpout exterior aft wall (Nod 2) (Checinska Sielaff et al., 2019; Urbaniak et al., 2022). Sampling details are as follows. MT1: 2015–2016; 3 flights; *n* = 24 wipes (Checinska Sielaff et al., 2019). MT2: 2017–2018; 5 flights; *n* = 40 wipes (Urbaniak et al., 2022). MT3: 2021–2022; 3 flights; *n* = 120 wipes, with five consecutive sampling days within each flight (Unpublished). The shotgun metagenomics datasets corresponding to MT1, MT2 and MT3 are available at NCBI Sequence Read Archive under the accession numbers PRJNA438545, PRJNA781277 and PRJNA1328788, respectively.

Metagenomics reads were searched for homologs of the 667 amino-acid putative MAO protein encoded by the *L. virtsii* F6-8S-P-1A lineage using DIAMOND blastx v0.9.14 (Buchfink et al., 2015). Initial MAO-like hits were retained using a permissive reporting threshold, followed by run-level classification into evidence categories. MAO homolog-positive runs required reads with ≥ 70% amino-acid identity, ≥ 25 aligned amino acids and e-value ≤ 1 × 10^−5^, together with ≥ 3 candidate read mates and ≥ 10% non-redundant coverage of the MAO query protein. Strong F6-8S-P-1A-like MAO-positive runs required close-hit reads with ≥ 90% amino-acid identity, ≥ 30 aligned amino acids and e-value ≤ 1 × 10^−10^, together with ≥ 5 close read mates and ≥ 50% non-redundant coverage of the MAO query. For positive runs, MAO-hit read pairs were extracted using SeqKit v2.12.0 (Shen et al., 2016) and taxonomically profiled with Kraken2 v2.1.3 (Wood et al., 2019) to determine the microbial context of the signal. Strong-positive read pairs were additionally mapped against the F6-8S-P-1A genome assembly using BWA v0.7.17-r1188 (Li and Durbin, 2010), and mapping statistics were calculated with SAMtools v1.9 (Li et al., 2009) to assess whether they mapped to the expected MAO-containing genomic locus. The initial run manifest comprised 355 SRA records, including 42 MT1, 64 MT2 and 249 MT3 runs. Since the numbers of available metagenomic runs differed in MT3, inter-dataset comparisons were based on the proportion (%) of MAO-positive runs within each dataset rather than absolute hit counts.

### 2.4 Growth kinetics and biofilm formation assays

Previous studies have used DA-supplemented LB to enhance the growth of *P. aeruginosa* PAO1 and *E. coli* (Xiang et al., 2024; Xu et al., 2026). Growth experiments were performed for *L. virtsii* and *L. williamsi* in citrate buffer (pH 5.6) (CB) and 1% (w/v) Tryptic Soy Broth (TSB, HiMedia, India) prepared in CB, with or without 1 mM DA input. For seed culture, cells were grown in 10 mL of TSB in a 50 mL Falcon tube and incubated at 37° for 24 h at 120 rpm. The culture was pelleted, washed twice with CB, and inoculated into 10 mL of media in a 25 mL capacity conical flask. Initial optical density (OD) was set to ~0.1 at 600 nm, which was further estimated at different time intervals by reading OD at 600 nm. Alternatively, cell suspensions were serially diluted, plated onto TSA, and incubated for 48 h at 37 °C to estimate viable cells, which were reported as colony-forming units (CFU) ml^−1^. The biofilm formation assay was carried out on cells grown in plain CB and in CB supplemented with 1 mM DA (CBD). Two hundred microliter of cultures (OD 0.1 at 600 nm) were added in triplicate to microwell plates and incubated for 72 h under static conditions, along with their respective cell-free blanks. Planktonic cells were estimated by reading the OD at 600 nm, as they are involved in colonization to new niches due to their free-floating motility. Biofilm formation was estimated by crystal violet (crystal violet) staining, followed by 33% acetic acid treatment, and absorbance was measured at 570 nm (Stepanović et al., 2007).

### 2.5 Assessment of DA metabolism using UV–visible spectrophotometry and microscopy

DA displays a strong absorption maximum around 280 nm in pure or acidic-to-neutral aqueous solutions, and a secondary peak can also be seen around 218 nm (Falamas et al., 2022). In contrast, the oxidized form of DA exhibits a broad absorption band around 390 nm (Farcas and Bende, 2024). Culture samples were assessed through UV–visible spectrophotometry to confirm the stability of DA in CB. The 3-day-old culture suspension was centrifuged, and the cell-free filtrate was analysed by UV spectrophotometry (230–400 nm). CB and CBD without inoculated cells served as blanks. Fluorescence measurements were taken to ascertain cell viability and increase in cell number. The cells were harvested and washed twice with 1X phosphate-buffered saline (PBS, pH 7.4). The pellet was then incubated with 100 μL PBS, 50 μL 3:7 acridine orange solution and 50 μL ethidium bromide for 30 min in the dark. Pellets were washed with PBS and observed under a fluorescence microscope (Zoe, Bio-Rad). Cells adhered to a sterile carbon tape placed in CB and CBD for 48 h were fixed, sputtered with a 5 nm gold layer and observed under a field emission scanning electron microscope (JEOL-JSM5800LV, Japan) (Fischer et al., 2024).

### 2.6 Colorimetric analysis of bacterial dopamine metabolism, media pH, extracellular proteins and NH_4_^+^ secretion

DA metabolism in *L. virtsii* and associated variation in pH were traced using alamar blue and phenol red as indicators, respectively. One hundred microliter of 3-day-old CB and CBD culture suspensions were treated with 10% (v/v) alamar blue reagent and incubated at 37 °C for 30 min. The absorbance was measured at 570 and 600 nm using a microplate reader (FLUOstar Omega, Germany), and the percentage reduction of alamar blue dye was measured according to the manufacturer’s protocol, as previously described (Sonam et al., 2024). The change in media pH was determined by adding 10 μL of 10% (v/v) phenol red to 100 μL of cell-free supernatant. The absorbance was measured immediately at 560 nm using a microplate reader (FLUOstar Omega, Germany) to determine the changes in media alkalinity.

Variation in total extracellular proteins and NH_4_^+^-N in *L. virtsii* as a function of DA metabolism were estimated using the Bradford method (Hammond and Kruger, 1988) and Baethgen and Alley’s (1989) principle (Rekha et al., 2020), respectively. Bovine serum albumin and (NH_4_)_2_SO_4_ were used as standards for estimating total protein and NH_4_^+^-N, respectively. For protein estimation, 50 μL of the sample was mixed with 100 μL of Bradford reagent (HiMedia, India) in a microplate, incubated for 30 min at room temperature and absorbance was measured at 590 nm in a microplate reader (FLUOstar Omega, Germany). For NH_4_^+^-N estimation, aliquots (100 μL) of the cell-free culture sample was mixed with 50 μL sodium salicylate-sodium nitroprusside solution (15%:0.03%), followed by 50 μL sodium hypochlorite (0.32%) and incubated for 30 min at room temperature. The absorbance was read at 650 nm in a microplate reader (FLUOstar Omega, Germany).

### 2.7 Assessment of impact of NH₄^+^ ions on media pH under abiotic conditions

A colorimetric assay was performed to investigate the variation in pH due to the addition of NH_4_^+^ ions to liquid CB (pH 5.6) and full-strength Nematode Growth Medium (NGM, pH 6.3). CB and NGM media were supplemented with 0–100 mM NH_4_^+^, transferred to a microplate and mixed with 10 μL of phenol red reagent. The absorbance was measured at 560 nm using a microplate reader (FLUOstar Omega, Germany). The pH of samples was measured using a digital pH meter (Systronics, India).

### 2.8 MAO gene expression analysis through real-time quantitative reverse transcription PCR (qRT-PCR)

The time course of the effect of DA on MAO expression was studied through qRT-PCR using the 16S rRNA gene as a reference. Cells of *L. virtsii* were cultured in 100 mL of CB and CBD with an initial OD of 0.1 and incubated (37 °C, 120 rpm, 48 h). The culture suspension was pipetted out periodically (4, 20, 28, and 48 h), centrifuged (8,000 rpm, 10 min, 25 °C), and total RNA was extracted from the cell pellets using RNAiso Plus (Takara) according to the manufacturer’s protocol. Contaminating residual DNA was removed by treating total RNA (20 μL) with 10 μL of 1 mg/mL DNase I (SRL) at 37 °C for 20 min, followed by a thermal inactivation (65 °C) for 10 min. Removal of contaminating DNA was verified through a 35-cycle PCR specific to the 16S rRNA gene using DNA polymerase and species-specific 16S rRNA primers (Table S3). The extracted RNA was quantified and used as a template for complementary DNA (cDNA) synthesis using the PrimeScript 1st Strand cDNA Synthesis Kit (Takara). Real-time PCR was performed with a SYBR kit (Takara). The details of MAO (test) and 16S rRNA gene primers used for qRT-PCR are given in Table S3. qRT-PCR was performed with an initial denaturation at 95 for 5 min, followed by 35 cycles of 95 °C for 30 s, 55 °C for 30 s and 72 °C for 30 s. Relative gene expression was calculated using ΔΔC_t_ to determine the fold-change in gene expression (Zhou et al., 2022).

### 2.9 Comparative analysis of gene expression through reverse transcription PCR (RT-PCR) and densitometry

The gene expression patterns of MAO, DNA gyrase B (*gyrB*), elongation factor Tu (*tuf*) and cellulose synthase-like protein (*cslA*), respectively involved in DA catabolism/NH_4_^+^ formation, DNA replication and transcription, translation and biofilm formation, were investigated through RT-PCR. A gene encoding RNA polymerase sigma factor (*rpoD*) was used as a reference. Cells of *L. virtsii* were cultured in 100 mL of CB and CBD for 48 h, culture suspension was retrieved at 4, 24, and 48 h and processed immediately for total RNA extraction using RNAiso Plus (Takara) according to the manufacturer’s protocol. The contaminating residual DNA was removed and cDNA was synthesized using a kit-based method as described above. The details of primers used for the amplification of *gyrB, tuf, MAO, cslA* and *rpoD* from cDNA template (2 μL each, except for *cslA*, where only 5 μL cDNA template was used) of *L. virtsii* are summarized in Table S3. The PCR conditions were as follows: 95 °C for 3 min, and then 40 cycles of 95 °C for 30 s, 65 °C for 30 s, and 72^o^ C for 30 s and final extension at 72 °C for 8 min. The DNA bands appeared on agarose gel was quantified using ImageJ (Tomlinson et al., 2024). The intensity profiles of individual lanes were generated by plotting the intensity peaks, while corresponding areas under each peak were measured (Vengili et al., 2021; Tomlinson et al., 2024). The band intensities were quantified for *gyrB, tuf, MAO* and *cslA* and divided by the corresponding *rpoD* intensity value for each sample to normalize the data and express the values as fold-change.

### 2.10 *In vivo* analysis of DA catabolites for modulation of cognition and chemotaxis

The ability of bacterial DA catabolites to influence chemotaxis and cognition in *C. elegans* was tested through the following assay systems: (1) pre-starvation and chemotaxis (conditioning stimulus); (2) preconditioning and chemotaxis (associative learning); (3) chemotaxis without pre-starvation and preconditioning. Appropriate controls (labelled as none) were maintained for all three sets of experiments for statistical analysis. *C. elegans* was maintained *in vitro* on NGM seeded with *E. coli* OP50 at 16 °C (Porta-de-la-Riva et al., 2012). The adult stages of *C. elegans* were synchronized using M9 buffer and a 20% bleaching solution (Bajire et al., 2023). Eggs were transferred to a fresh NGM plate containing an *E. coli* OP50 lawn and incubated at 16 °C for 4–6 days to harvest adults.

*C. elegans* responds to acute starvation by arresting development and altering gene expression and metabolism at various phases of the life cycle (Baugh and Hu, 2020). Starvation is a conditioning stimulus influencing chemotaxis in *C. elegans* (Saeki et al., 2001). Adult worms were starved for 1 h before being placed on NGM plates to facilitate a rapid chemotactic response. 50 mm NGM plates were marked with four quadrants and inoculated with *E. coli* OP50 suspension, CB, CBD, and secretome of *L. virtsii* grown in CBD (SUP) (10 μL each). 1 h starved *C. elegans* (*n* = 30) were placed at the center of the plate and the movement of the worms was recorded (Crombie et al., 2022). Similarly, motility of 1 h starved worms (*n* = 30), placed at the centre of a plain 50 mm NGM plate without *E. coli* OP50 or chemical cues, served as controls.

Further, a chemotaxis assay was designed to study the impact of pre-conditioning on the cognition and motility of *C. elegans*. In order to understand the associative learning, the worms (*n* = 30) were pretreated with *E. coli* OP50, CB, CBD and SUP for 60 min and placed at the center of plates marked with four quadrants, which were drop-coated with *E. coli* OP50, CB, CBD and SUP (10 μL). Control worms (*n* = 30), pre-treated with CB, CBD, *E. coli* OP50 culture and SUP for 60 min, were placed at the centre of a 50 mm bare NGM plate and tracked for their migration towards empty quadrants. The chemotaxis indices were measured at two time points.

Finally, *C. elegans* chemotaxis towards NH₄^+^ vs. CB/SUP was investigated without pre-starvation and pre-conditioning. The chemotaxis indices of worms were measured on agar quadrants drop-coated with CB and SUP. The indices were also measured separately on agar quadrants drop-coated with 0–50 μM NH_4_^+^ vs. CB and SUP. Appropriately diluted aqueous NH_4_OH (10 μL) was drop-coated on NGM plates. Worms (*n* = 30) were introduced at the center of the plate and the movement was recorded at regular intervals and the chemotaxis index was calculated (Marogi et al., 2024; Neto et al., 2016). Control worms (*n* = 30) were placed at the centre of 50 mm bare NGM plates and tracked their migration towards empty quadrants.

### 2.11 Statistical analysis and software

For metagenomic comparisons, an MAO-positive run was defined as a run meeting either the candidate MAO homolog-positive or strong F6-8S-P-1A-like MAO-positive criteria defined in Section 2.3; all other screened runs were considered negative. Differences in MAO-positive proportions among MT1, MT2 and MT3 were assessed using two-sided pairwise Fisher’s exact tests on 2 × 2 contingency tables, with Benjamini–Hochberg correction for the three pairwise comparisons. Adjusted *p* < 0.05 was considered statistically significant. All metagenomic statistical analyses were performed in R version 4.5.1. Statistical significance *in vitro*, *in vivo*, abiotic pH assays and densitometric scans was determined using ordinary one-way ANOVA with Tukey’s multiple comparison tests using GraphPad Prism. One-way ANOVA for the chemotaxis assay data was done using Tukey’s multiple comparisons test for starvation and associated chemotaxis assay of *C. elegans*. Two-way ANOVA was performed for the chemotaxis after pre-conditioning using Sidak’s multiple comparisons test. Mixed-effects analysis for starvation- and preconditioning-free chemotaxis assay data generated for CB vs. SUP, CB vs. 0–50 μM NH_4_^+^ and SUP vs. 0–50 μM NH_4_^+^ was based on Bonferroni’s multiple comparisons test. Chemical structures were drawn using RCSB.org (https://www.rcsb.org/chemical-sketch). The ImageJ (Tomlinson et al., 2024) tool was adopted for densitometry of RT-PCR products.

## 3 Results

### 3.1 Heterogeneous occurrence of potential dopamine catabolism in *Leifsonia*

The schematic representation of the enzymatic transformation of DA to homovanillic acid (HVA), deduced through the literature survey (Mannisto and Kaakkola, 1999; Meiser et al., 2013), possibly occurring in *L. virtsii*, is shown in Figure 1a. *L. virtsii* harboured a complete set of genes encoding MAO, COMT and ALDH dedicated to DA degradation. MAO-producing *L. virtsii* was chosen for detailed investigation since MAO catalyzes the first step of DA catabolism, yielding 3,4-dihydroxyphenylacetaldehyde (DOPAL), NH_3_ and H_2_O_2_. In addition, MAO can also transform 3-methoxytyramine into 4-hydroxy-3-methoxyphenylacetaldehyde (homovanillin), yielding NH_3_ and H_2_O_2_ (Figure 1a). While NH₃ is inherently a membrane-permeable gas and potent neurotoxin (Gaidin et al., 2020), NH₄^+^ is an ionic species formed due to the spontaneous reaction of NH_3_ with water and exerts fundamentally different biological effects. The detection of MAO in *L. virtsii* reflects its potential ability to yield NH₃/NH₄^+^ through neurotransmitter metabolism.

**Figure 1:**
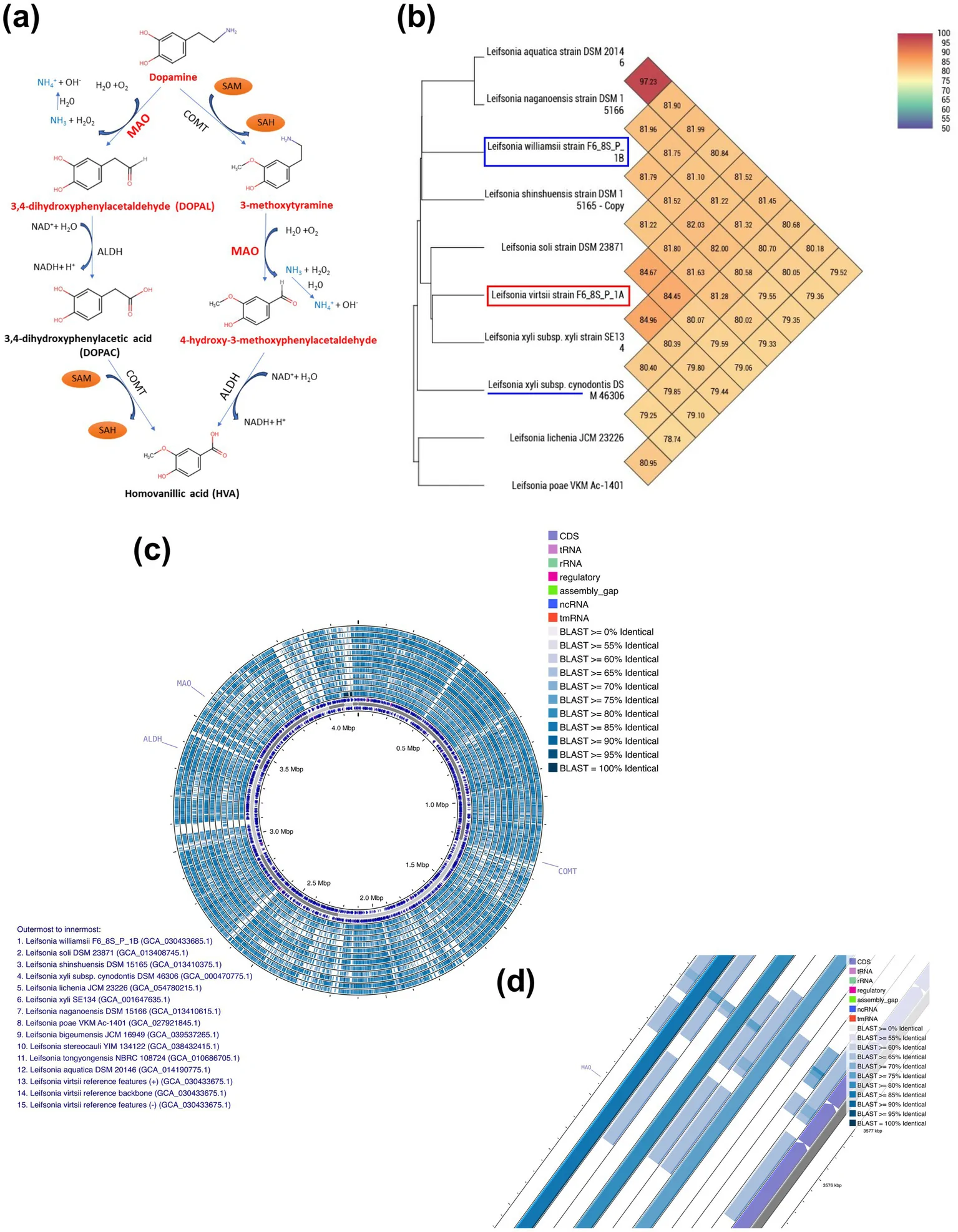
Genomic potential for dopamine (DA) degradation in ISS isolate *Leifsonia virtsii* F6_8S_P_1A^T^. Putative DA degradation pathway mediated by monoamine oxidase (MAO), catechol-O-methyltransferase (COMT) and aldehyde dehydrogenase (ALDH) detected in *L. virtsii* **(a)**. OrthoANI heatmap showing the genomic relatedness of *L. virtsii* (red box) with *L. williamsii* (blue box) and other closely related type strains (based on EzBiocloud 16S rRNA gene search) **(b)**; all species harboured MAO, except for *L. williamsii* and *L. xyli* ssp. *cynodontis* (blue underline). Comparative circular genome map of the focal ISS isolate *L. virtsii* and 12 validly established type strain genomes of *Leifsonia* **(c)** and a zoom in view showing the gene encoding MAO **(d)**. The *L. virtsii* genome is the reference and is shown innermost. Blue shading shows nucleotide similarity to the reference, with darker blue indicating greater identity. MAO, ALDH, and COMT are labelled on the reference genome.

*Leifsonia* species that shared high 16S rRNA gene sequence similarity (>97.9%) and OrthoANI values (79.44–84.96%) with *L. virtsii* were screened for genes encoding MAO, ALDH, and COMT. All target genes were present in *Leifsonia* species, except for *L. williamsii* and *L. xyli* ssp. *cynodontis* that lacked MAO (Figure 1b). We found only 14 species validly placed under the genus *Leifsonia* in LPSN (Supplementary Table S1), while 8 species have been reclassified into 7 distinct genera within the phylum Actinomycetota (Supplementary Table S2). MAO was found in 61.5% (8/13) of validly established genome-sequenced *Leifsonia* (Figures 1c,d; Supplementary Table S1), while ALDH-COMT was absolute in occurrence. Interestingly, MAO was also found in 62.5% (5/8) of reclassified *Leifsonia* species (Supplementary Table S2). These data indicated the heterogeneous dispersal of MAO in authentic *Leifsonia* and justified the selection of *L. williamsii* as a MAO-negative control for direct comparative analysis.

Phylogenetic analysis was carried out to understand the congeneric distribution of MAO within the phylum Actinomycetota and to trace the closest phylogenetic neighbours. The maximum likelihood phylogeny revealed a distinct lineage formed by *L. virtsii* MAO within the clade that accommodated type strains of *L. soli*, *L. lichenia* and *L. poae,* respectively, isolated from teak tree rhizosphere soil (Madhaiyan et al., 2010), lichen (An et al., 2009) and annual meadow grass (Evtushenko et al., 2000) (Supplementary Figure S1). This is in line with the pangenomic data, where high BLAST scores were obtained for corresponding species vs. *L. virtsii* (Figures 1c,d). The phylogeny supported the neighbourhood of ISS isolate *L. virtsii* to Earth-bound *Leifsonia*, besides highlighting congeneric heterogeneous dispersal of MAO homologs across genetically diverse Actinomycetota.

### 3.2 Detection of MAO signature in ISS metagenome and evidence for temporal enrichment

Metagenomics data of NASA corresponding to three microbial tracking missions (MT3), spanning a period of 7 years, were screened for the possible occurrence, temporal variations and taxonomic identity of MAO in ISS. In total, 355 ISS metagenomes with available FASTQ data were screened across MT1, MT2 and MT3. Raw MAO-like DIAMOND hits were detected in 323/355 metagenomes, indicating that distant MAO-like homologs could be common in the ISS metagenome. After applying read and coverage thresholds, 85 metagenomes retained evidence of MAO homologs related to the query *L. virtsii*. Of these, 83 were classified as MAO homolog-positive runs, whereas two metagenomes met the stricter criteria for a strong *L. virtsii*-like MAO signal. Of these, 83 were classified as broad candidate *L. virtsii* MAO homolog-positive runs, while only two metagenomes met the stricter criteria for a strong *L. virtsii*-like MAO signal (Figure 2a). MAO positive frequencies were found to be 4.8% (2/42) in MT1, 12.5% (8/64) in MT2 and 30.1% (75/249) in MT3. Pairwise Fisher’s exact tests with Benjamini–Hochberg correction showed significantly greater MAO-positive frequency in MT3 compared with MT1 (adjusted *p* = 0.000694) and MT2 (adjusted *p* = 0.00606), whereas MT1 and MT2 did not differ significantly (adjusted *p* = 0.309). These two runs represented matched non-PMA and PMA-treated libraries from the Node 2 Port crew quarters exterior aft wall sampled on 6 February 2018, corresponding to the same ISS location and sampling context as the original isolate *L. virtsii*. They showed 41 candidate read mates covering 489/667 amino acids of the query protein, corresponding to 73.31% candidate coverage, and 10 close read mates covering 416/667 amino acids, corresponding to 62.37% close-hit coverage. SRR17140484 showed 88 candidate read mates covering 650/667 amino acids, corresponding to 97.45% candidate coverage, and 29 close read mates covering 602/667 amino acids, corresponding to 90.25% close-hit coverage. Both strong-positive runs contained reads with 100% amino-acid identity to the MAO query. Kraken2 profiling of extracted MAO-hit read pairs showed that the signal was taxonomically structured and predominantly associated with Actinomycetota*-*related genera. Among the 83 candidate-only runs, 63 yielded a genus-level top assignment. Together, *Micrococcus* and *Leifsonia* represented 30/63 classified candidate-only runs (47.6%), while most of the remaining were found to be affiliated to the other genera placed under phylum Actinomycetota. In the two strong-positive MT2 libraries, extracted MAO-hit read pairs were assigned mainly within Microbacteriaceae, with strong *Leifsonia*-associated assignments (Figure 2b), and read recruitment placed the strong-positive reads at the same MAO-containing locus in the *L. virtsii* genome assembly. These data authenticated the ISS origin of *L. virtsii* and provided evidence for the temporal enrichment of MAO bearing Actinomycetota.

**Figure 2:**
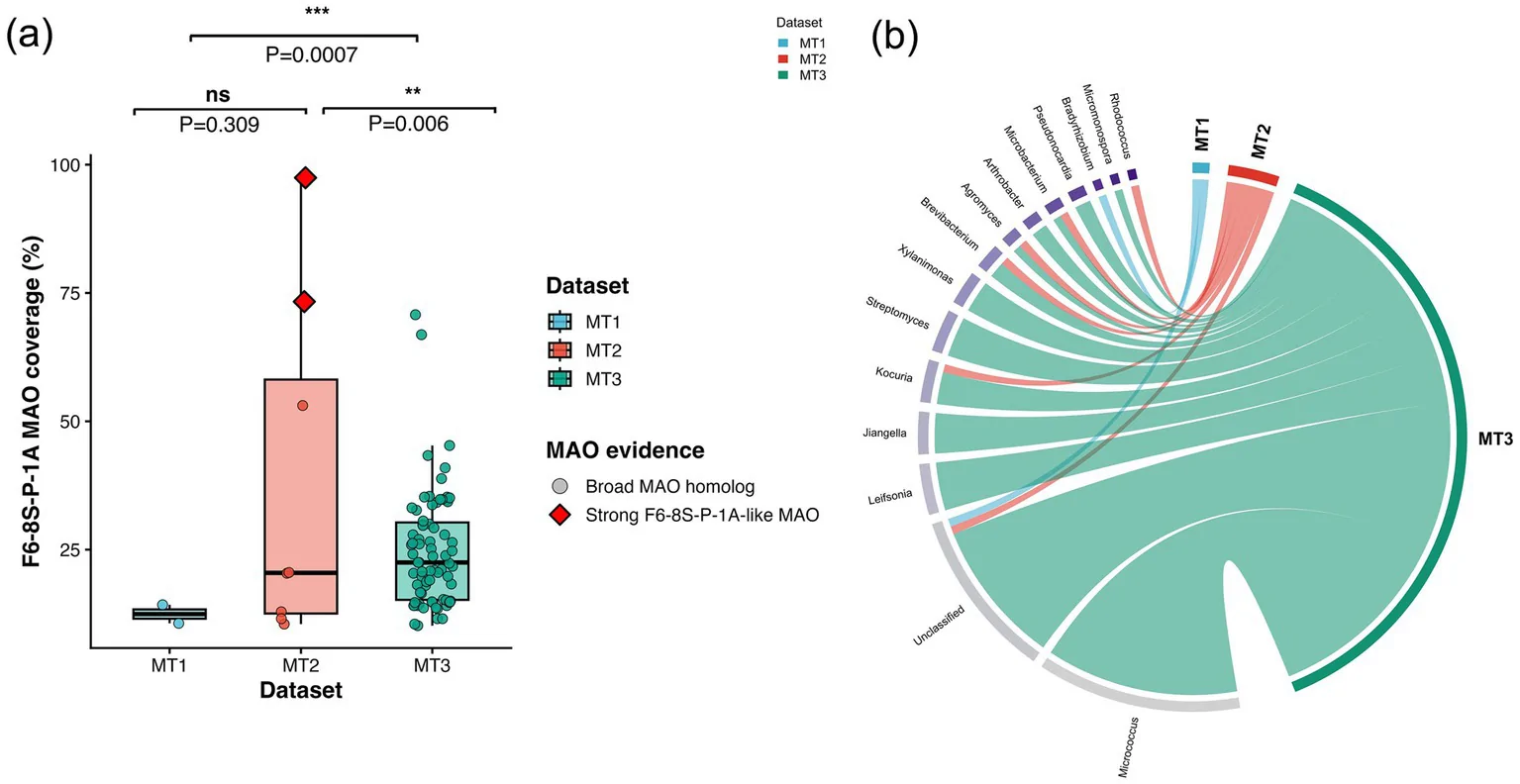
Distribution and taxonomic context of F6-8S-P-1A-like MAO signals across ISS Microbial Tracking metagenomes. Coverage of the 667-aa F6-8S-P-1A putative monoamine oxidase (MAO) protein across MAO-positive ISS metagenomes from MT1, MT2 and MT3 **(a)**. Boxplots show the distribution of MAO protein coverage per dataset, with individual SRRs overlaid. Grey circles indicate broad MAO homolog-positive metagenomes, while red diamonds indicate strong F6-8S-P-1A-like MAO signals. Strong F6-8S-P-1A-like signals were detected in two MT2 libraries. Chord diagram linking MAO-positive SRRs, grouped by MT dataset, to the dominant Kraken2 genus assigned to extracted MAO-hit read pairs **(b)**. Link width represents the number of MAO-positive SRRs assigned to each genus, and link colour indicates the MT dataset. The broader candidate MAO signal was dominated among classified assignments by Actinomycetota-associated genera, particularly *Micrococcus* and *Leifsonia*. Pairwise comparisons were performed using Fisher’s exact tests with Benjamini–Hochberg correction. Significance levels: ns, *p* > 0.05; **, *p* ≤ 0.01; ***, *p* ≤ 0.001.

### 3.3 DA stimulates cell division, viability, and biofilm formation in *Leifsonia virtsii*

We carried out *in vitro* assays to understand *L. virtsii*’s growth and metabolic response in DA-supplemented CB (pH 5.6) and DA-supplemented 1% (w/v) TSB prepared in CB (pH 5.6). Since NH_4_^+^ was one of the target nitrogenous metabolites, potentially formed from DA and its derivative 3-methoxytyramine (Figure 1a), bacterial culture experiments were carried out in CB of pH 5.6 in which NH₃/NH₄^+^ equilibrium (pKa ≈ 9.25) is > 99.99% in favor of NH₄^+^. We used CB (pH 5.6), which served as an excellent buffer for 1–10 mM NH_4_^+^ as evident under abiotic conditions (Supplementary Figures S2a,c). The growth curve obtained for *L. virtsii* in CB and 1% (w/v) TSB, with and without DA supplement, revealed increased growth in the presence of DA (Figure 3a). Cell counting revealed a high number of viable cells of *L. virtsii*, particularly when grown in CBD (Figures 3b,c). In contrast, *L. williamsii* grew slowly, and no significant growth difference was found between its CB and CBD cultures. These data indicated a stimulatory impact of DA on cell growth of *L. virtsii*.

**Figure 3:**
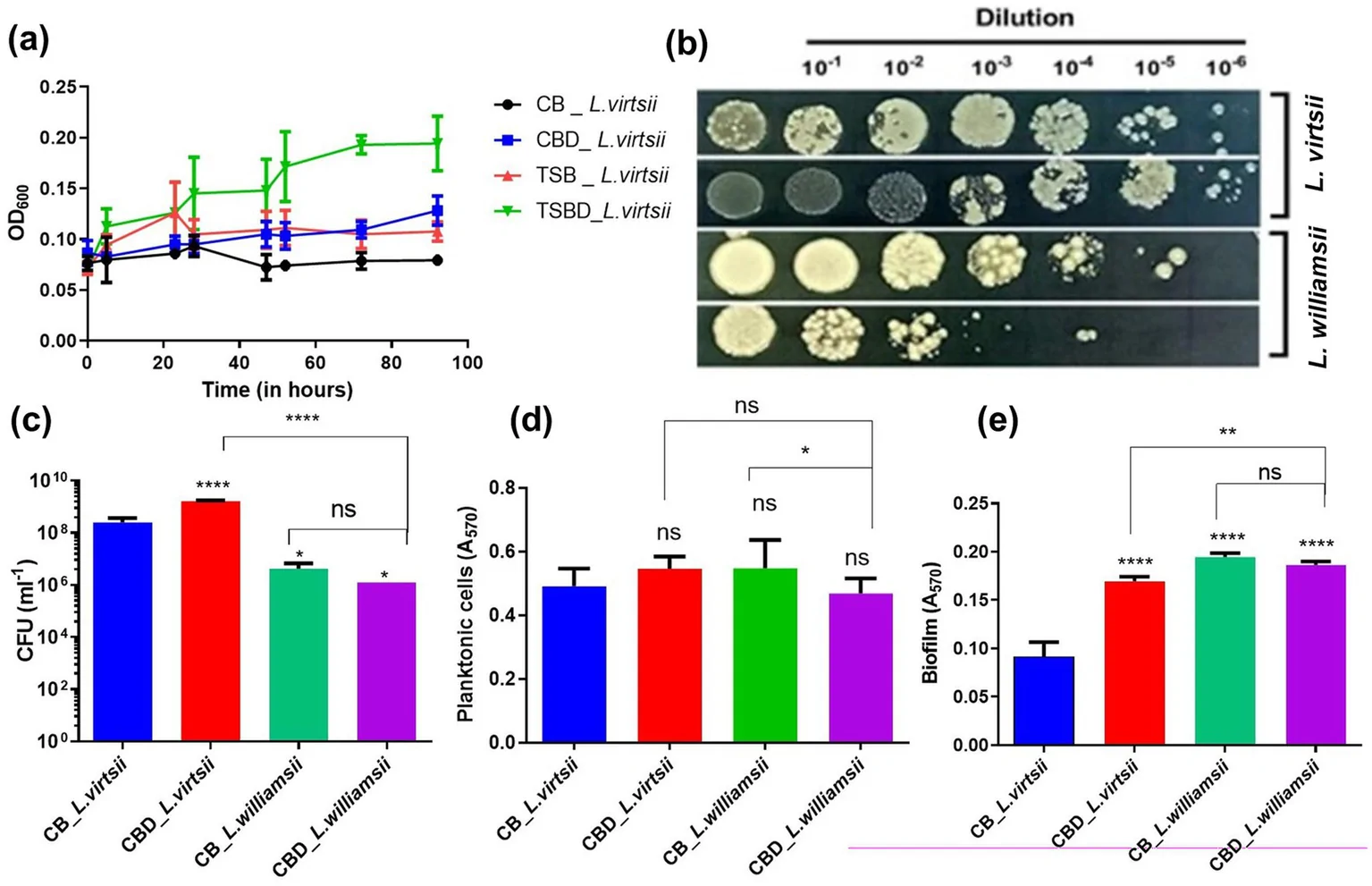
Growth kinetics, cell viability and biofilm formation in *Leifsonia virtsii* F6_8S_P_1A^T^ under dopamine (DA) supplementation. Growth curve obtained for *L. virtsii* in citrate buffer (pH 5.6) (CB) and 1% tryptic soy broth (prepared in CB) in the presence or absence of 1 mM DA **(a)**. Error bar, mean (*n* = 6) ± SD. Viability and cell count of *L. virtsii* and *L. williamsii* precultured in CB (upper panel) and CBD (lower panel) for 3 days were determined on TSA (**b,c**, respectively). Planktonic cells **(d)** and biofilm formation **(e)** in *L. virtsii* and *L. williamsii*, when cultured in the presence and absence of DA for 72 h, are shown. Biofilm formation was measured using crystal violet staining. Statistical comparisons were made using one-way ANOVA followed by Tukey’s multiple comparisons test. Error bar, mean (*n* = 3) ± SD and *p < 0.1, **p < 0.05, ***p < 0.01, ****p < 0.0001; ns, non-significant.

The impact of exogenous DA on biofilm formation was investigated using static microplate culture experiments. No significant difference in terms of planktonic cells was observed in *L. virtsii* maintained in CB/CBD, while *L. williamsii* showed a decline in planktonic cells when treated with CBD (Figure 3d). This suggests the negligible impact of DA on free-floating motility of *L. virtsii*. Biofilm formation was significantly higher in *L. virtsii* in the presence of DA (Figure 3e), while *L. williamsii* was found to be an intrinsically good biofilm former and DA had no positive impact on its biofilm formation.

Spectrophotometric and microscopic analyses were performed to validate the DA degradation and assess its impact on cell growth, viability and biofilm formation in *L. virtsii* (Supplementary Figure S3). The UV–visible spectrum of CBD showed a signature λ_max_ at 280 nm and a secondary peak at 214 nm, specific for DA (Supplementary Figures S3a,b). On the other hand, a tiny peak appeared in the CB pre-inoculated with *L. virtsii*, while no such peak was recorded for CB, suggesting it could be a bacterial metabolite. The absorbance of the primary peak decreased in CBD inoculated with *L. virtsii*, with no change in the secondary peak, suggesting DA degradation. In contrast, a decline in the absorbance of the primary peak of CBD inoculated with *L. williamsii*, accompanied by a concomitant increase in the secondary peak, indicated a possible transformation of DA rather than its degradation. A broader peak was observed at 330 nm when *L. williamsii* was cultured in CB, suggestive of a distinct bacterial metabolite. These data indicated DA degradation in *L*. *virtsii* and alternative formation of citrate-based metabolites in *L. williamsii*. Fluorescence and transmission electron microscopy were carried out to gain further insights into changes that occurred at the cellular level as a function of DA supplementation. Significantly high fluorescence encountered in *L. virtsii* cultured in CBD, substantiated cell growth and viability (Supplementary Figures S3c–g), and scanning electron microscopy validated the biofilm formation by *L. virtsii* (Supplementary Figure S3h).

### 3.4 DA metabolism results in the extracellular NH_4_^+^ secretion and media alkalization

MAO-based transformation of DA to DOPAL and/or 3-methoxytyramine to 4-hydroxy-3-methoxyphenylacetaldehyde (homovallin) results in the formation of NH_3_ (Figure 1a), which may change media pH due to the spontaneous formation of NH_4_^+^ ions. DA catabolites, on the other hand, may sustain the growth of MAO-bearing bacteria, influencing the extracellular enzymes/proteins. Thus, the impacts of DA on cellular metabolism, media pH, extracellular protein secretion, and NH_4_^+^ formation in *L. virtsii* were investigated and the results are shown in Figures 4a–d. *L. virtsii* in CBD showed a significant increase in alamar blue dye reduction (Figure 4a), media alkalization (Figure 4b), and extracellular NH_4_^+^-N (Figure 4d) compared with CB. Significantly high media alkalization (Figure 4b), extracellular proteins (Figure 4c) and NH_4_^+^ formation (Figure 4d) was found in CBD inoculated with *L. williamsii*; however, no specific increment in alamar dye reduction was recorded, suggesting a possible alternative pathway for NH_4_^+^ formation. Concurrent AB dye reduction and detection of NH_4_^+^-N suggested active DA metabolism in *L. virtsii*.

**Figure 4:**
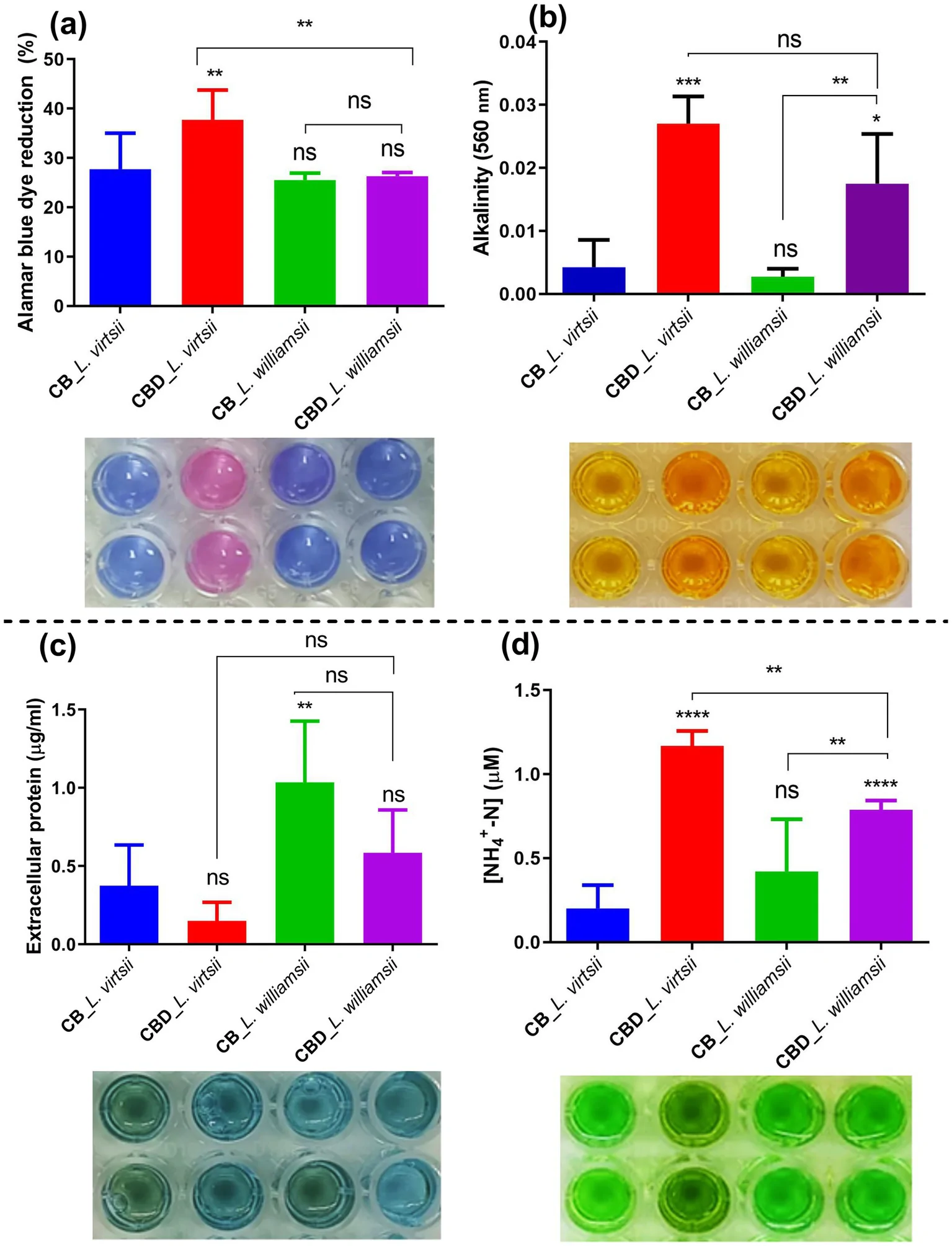
Impacts of DA on cellular metabolism, media pH, extracellular protein secretion and NH_4_^+^ formation in *Leifsonia virtsii* F6_8S_P_1A^T^. The response of *L. virtsii* and *L. williamsii* in the presence and absence of DA with regard to alamar blue dye reduction **(a)**, media alkalization **(b)**, extracellular protein secretion **(c)** and NH_4_^+^ formation **(d)** are shown. The sections of microplates representing alamar blue dye reduction, media alkalization, extracellular protein estimation and NH_4_^+^-N are shown. Statistical comparisons were made using one-way ANOVA followed by Tukey’s multiple comparisons test. Error bar, mean (*n* = 6) ± SD and *p < 0.1, **p < 0.05, ***p < 0.01, ****p < 0.0001; ns, non-significant.

### 3.5 Differential regulation of genes involved in dopamine catabolism/NH_4_^+^ formation, DNA replication/transcription, translation and biofilm formation under DA input

At first, the time course of the expression of a gene encoding MAO (*MAO*) and associated NH_4_^+^ formation in *L. virtsii*, grown in CBD, was investigated through colorimetric assay and qRT-PCR, respectively, using cells grown in CB as a control. The line diagram of NH_4_^+^ discharge kinetics after blank subtraction is depicted in Figure 5a. The representative sections of microplates showing the time course of NH_4_^+^ formation in CB and CBD in the presence and absence of *L*. *virtsii* are shown in Figure 5b. Rapid exponential increase in NH_4_^+^ were found when *L*. *virtsii* were grown in the presence of DA. qRT-PCR revealed a significant decline in MAO relative expression within 20 h, reaching complete inhibition of gene expression within 48 h of incubation (Figure 5a). These data suggested a possible declining trend in MAO expression with increasing NH_4_^+^ accumulation.

**Figure 5:**
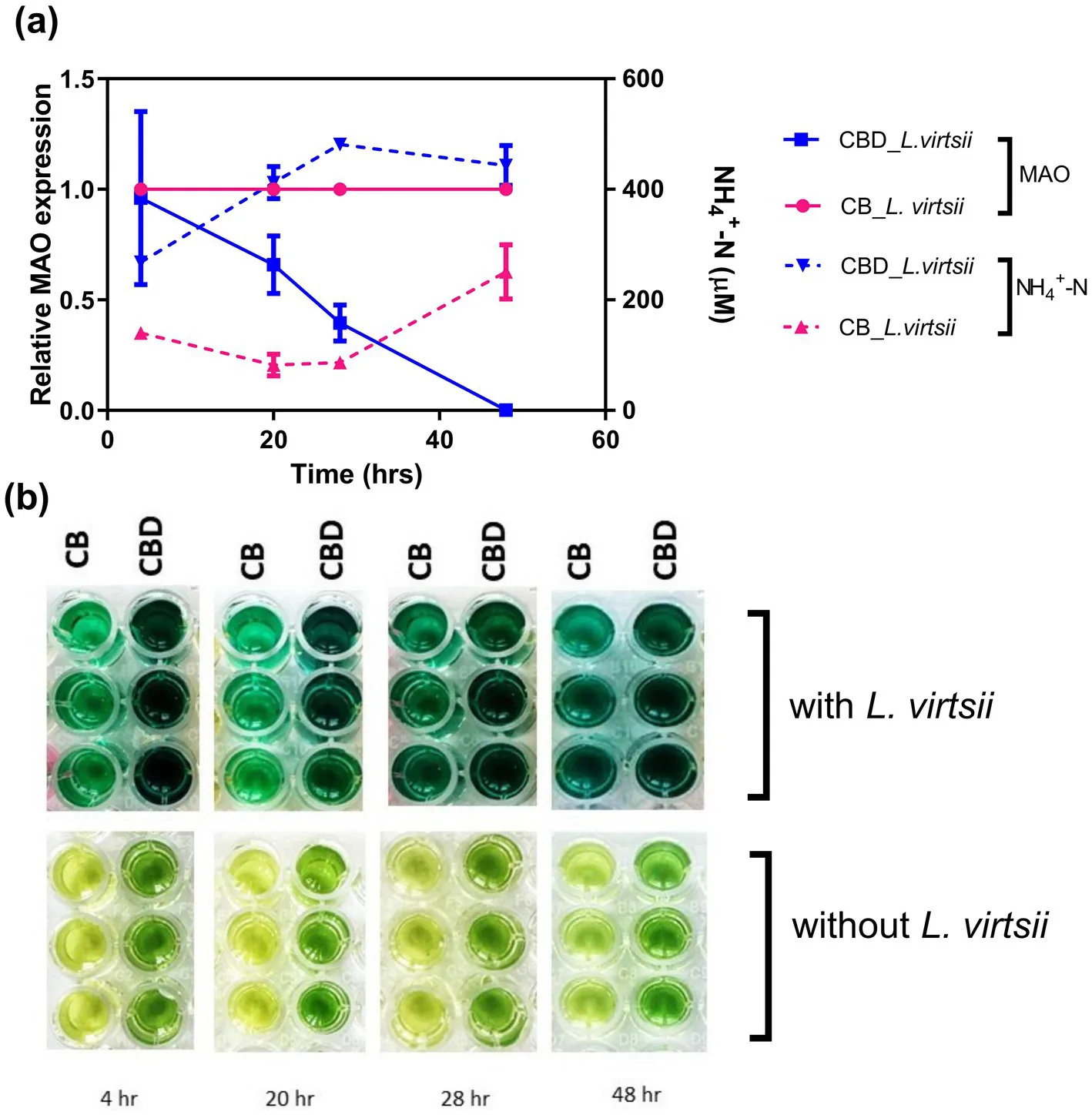
Dopamine (DA)-driven NH_4_^+^ formation in *Leifsonia virtsii* F6_8S_P_1A^T^ and MAO gene expression kinetics. Line diagram depicting the time course of MAO gene expression and associated NH_4_^+^ formation in *L. virtsii* at 4, 20, 28, and 48 h incubation in citrate buffer (pH 5.6) with and without DA supplementation (CB and CBD, respectively). The 16S rRNA gene was used as a reference gene **(a)**. Blank-subtracted values are taken to consideration to depict the trend in NH_4_^+^ formation. Fold-change in MAO gene expression is shown on the left Y-axis. NH_4_^+^-N (μM) is shown on the right Y-axis. Statistical comparisons were made using one-way ANOVA followed by Tukey’s multiple comparisons test. Error bars, MAO: mean (*n* = 3) ± SD; NH_4_^+^-N: mean (*n* = 6) ± SD. *p < 0.1, **p < 0.05, ***p < 0.01, ****p < 0.0001; ns, non-significant. Microplate sections show chromogenic reaction by *L. virtsii* incubated in CB and CBD for 4, 20, 28 and 48 h **(b)**.

The suppression in *MAO* expression in *L. virtsii* could be due to DA depletion and/or general growth phase-dependent transcriptional regulation unrelated to DA metabolism/associated NH_4_^+^ formation. To test this, time-course (4, 24, and 48 h) RT-PCR was performed, followed by densitometry targeted to *MAO*, *gyrB, tuf* and *cslA*, which represented DA catabolism/NH_4_^+^formation, DNA replication and transcription, translation and biofilm formation. The RNA polymerase sigma factor gene (*rpoD*) was used as a reference. Under DA input, *gyrB* exhibited an increasing trend expression, reaching a peak at 48 h, while its expression was poor in the absence of DA (Supplementary Figures S4, S5a). Similarly, DA supplementation resulted in significantly higher expression of the *tuf* and *cslA* at 48 h (Supplementary Figures S4, S5b,d). It is noteworthy that significant increments in *tuf* and *cslA* were also observed in bare CB, suggesting possible metabolism of basal citrate by *L. virtsii* to sustenance. RT-PCR showed significantly high MAO expression in *L. virtsii* within 4 h of DA exposure, whereas MAO expression peaked at 24 h in plain CB (Supplementary Figures S4, S5c). The upregulation of *gyrB, tuf,* and *cslA* may explain increased growth, metabolism and biofilm formation in *L. virtsii* under DA supplementation (Figure 3, 4). The rapid activation of MAO upon DA supplementation and its eventual decline suggest collective involvement of growth-phase-dependent transcriptional regulation and negative feedback by NH_4_^+^. Further studies are warranted to understand the enzyme kinetics and estimate specific activity using recombinant or purified MAO of *L. virtsii*.

### 3.6 NH_4_^+^, a DA catabolite modulates cognition and chemotaxis in *C. elegans*

The impacts of DA catabolites on cognition and chemotaxis were investigated using *C. elegans* as an *in vivo* model. Since chemotaxis at alkaline pH was reported in *C. elegans* (Murayama and Maruyama, 2013), behavioural experiments in *C. elegans* were conducted on NGM (pH 6.3), which served as an excellent buffer for the tested doses (0–50 μM) of NH_4_^+^ (Supplementary Figures S2b, d). At first, chemotaxis of starved *C. elegans* was tested on NGM agar quadrants loaded with *E. coli* OP50, CB, CBD and CBD culture supernatants of *L. virtsii* (SUP). We found a significant positive chemotaxis in *L. virtsii* towards *E. coli* OP50, suggestive of its inherent capacity to move towards *E. coli* OP50 as a choice of food (Figures 6a,b). The effects of preconditioning on the cognition and chemotaxis of *C. elegans* were investigated using agar quadrants loaded with *E. coli* OP50, CB, CBD, and SUP. *C. elegans* pre-treated with *E. coli* OP50, CB and CBD yielded a significant negative chemotaxis index towards the respective agents (Figures 6c–e). In contrast, pre-treatment with SUP resulted in significant positive chemotaxis of *C. elegans* specifically towards SUP (Figure 6f). This could reflect associative learning of a food cue (bacterial metabolites) in *C. elegans.*

**Figure 6:**
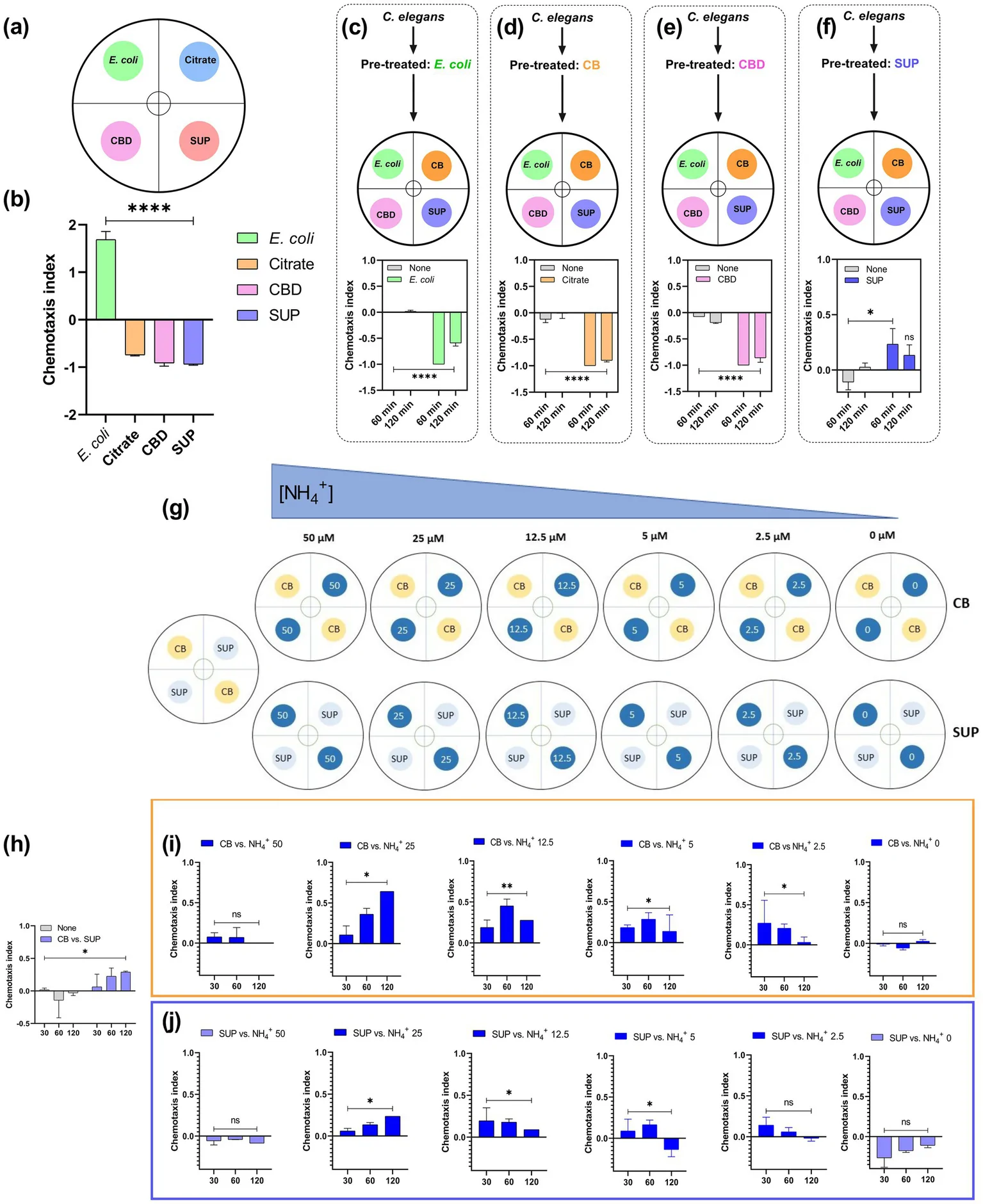
Assessment of impacts of DA catabolites on cognition and chemotaxis of *C. elegans*. Schematic view of the experimental setup to probe the sensory perception of starved worms **(a)**. Chemotaxis indices obtained for starved *C. elegans* when placed with *E. coli* OP50, citrate buffer (pH 5.6) (CB), CB containing 1 mM DA (CBD), and CBD supernatant of *L. virtsii* (SUP) **(b)**. One-way ANOVA for the chemotaxis data was performed with Tukey’s multiple comparisons test with *E. coli* OP50 as a control. The impacts of pre-treatment of test agents on the cognition and chemotaxis of *C. elegans* were investigated using agar quadrants loaded with *E. coli* OP50 **(c)**, CB **(d)**, CBD **(e)**, and SUP **(f)** are shown. Two-way ANOVA for the chemotaxis assay for pre-treated worms was done using Sidak’s multiple comparisons test; the migration of pre-treated worms (*n* = 30) placed at the centre of a plain NGM plate at the corresponding time point was taken as the control (specified as none). Schematic view of the experimental setup used for assessing the impacts of DA catabolite NH_4_^+^ on chemotaxis of *C. elegans* vs. CB and SUP **(g)**. Chemotaxis indices measured for *C. elegans* when exposed to CB and SUP, showing chemotaxis preference **(h)**. Chemotaxis indices measured for *C. elegans* when provided with 50, 25, 12.5, 5, 2.5, and 0 μM NH_4_^+^ vs. CB **(i)**. Chemotaxis indices measured for *C. elegans* when provided with 50, 25, 12.5, 5, 2.5, and 0 μM NH_4_^+^ vs. SUP **(j)**. Mixed-effects analysis for chemotaxis assay data generated for CB vs. SUP, CB vs. 0–50 μM NH_4_^+^ and SUP vs. 0–50 μM NH_4_^+^ was based on Bonferroni’s multiple comparisons test. The migration of worms (*n* = 30) placed at the centre of a plain NGM plate at the corresponding time point was taken as the control (specified as none). Error bar, mean (*n* = 30) ± SD. *p < 0.1, **p < 0.05, ***p < 0.01, ****p < 0.0001; ns, non-significant.

We further investigated the effects of CB and SUP on *C. elegans* chemotaxis. In addition, we also tested increasing doses of NH_4_^+^(0–50 μM), a DA catabolite, on the chemotaxis of *C. elegans* using agar quadrants loaded either with CB or with SUP as counter metabolite (Figure 6g). Supplementing 2.5, 25 and 50 μM NH_4_ resulted in 0, 0.03 and 0.18 increases in media pH, while SUP showed 0.08 increase in pH as compared to un-inoculated CBD. The preliminary quadrant assay revealed the significant positive chemotaxis of *C. elegans* towards SUP when CB was used as a counter metabolite (Figure 6h). Positive chemotaxis was recorded for *C. elegans* towards 2.5–25 μM NH_4_^+^, when provided with CB as a counter metabolite; however, chemotaxis was recorded to be low in 50 and 0 μM NH_4_^+^ suggesting a possible sensory threshold. Similarly, positive chemotaxis was recorded when supplied with 2.5–25 μM NH_4_^+^ along with SUP as a counter metabolite. However, interestingly, indices were relatively lower than those of the CB counterpart. This could be due to interference from alternative chemoattractant (including NH_4_^+^) present in SUP, interfering with the worm’s chemotaxis towards NH_4_^+^. Nonetheless, our findings highlight modulatory impacts of DA catabolites, particularly NH_4_^+^_,_ on *C. elegans* motility.

## 4 Discussion

The present study goes beyond the conventional description of microbial persistence associated with the ISS (Singh et al., 2018) and proposes a new framework connecting microbial neurotransmitter metabolism, influencing the behaviour of a eukaryotic host. Our results indicate that the ISS isolate *L. virtsii* has a specialized metabolic adaptation that allows it to metabolize DA, and exhibit growth and biofilm formation. The analysis based on 16S rRNA and the MAO genes, along with a phylogenomic study, indicated a significant genetic relatedness between *L. virtsii* and several terrestrial and aquatic species (Suzuki et al., 1999; Evtushenko et al., 2000; An et al., 2009; Madhaiyan et al., 2010). This investigation further emphasized the heterogeneous occurrence of DA-metabolizing capabilities prevailing among Actinomycetes species, which have been recovered from a range of ecological settings, including soil and space (Figures 1b–d, Supplementary Table S1). ClustalX and MEGA 5 have been widely used for molecular sequence analysis and multiple sequence alignment, respectively, and continue to be applicable for phylogenetic and comparative sequence analyses (Li et al., 2023; Martínez-Murcia et al., 2024). While comparative genomics and OrthoANI analysis provided taxonomic resolution, the maximum likelihood tree aided in the identification of the closest related phylogenetic neighbours based on the MAO sequences. The discovery of MAO signatures at ISS and Earth-bound counterparts offered new insights into the adaptability of Actinomycetes to nutritionally varied and spatially complex habitats.

Understanding the long-term effects of space travel on human biology is one of the objectives of researchers aboard the ISS (Kitmacher et al., 2005). A key novelty of this work is the identification of an MAO-based DA metabolic pathway in a novel ISS-associated bacterial species.

The metagenomics data show that MAO-like homologs are widespread across ISS metagenomes, whereas evidence for stringent *L. virtsii*-like MAO is rare. The proportion (%) of MAO-positive runs showed significantly greater prevalence of MAO in MT3 than in MT1 or MT2. The overall candidate MAO signal was taxonomically structured to Actinomycetota. The two strong MT2 signals corresponded to matched libraries from the same ISS surface sampling context as the *L. virtsii*: the Node 2 Port crew quarters exterior aft wall sampled on 6 February 2018, validating strain’s ISS origin (Simpson et al., 2023). Combined reads of these two libraries reached 100% amino-acid identity, broad MAO protein coverage, Microbacteriaceae/*Leifsonia-*compatible taxonomic support and recruitment to the expected MAO-containing genomic locus. They provide the strongest metagenomics evidence for an *L. virtsii*-like MAO signal prevailing in ISS metagenomes. The present MAO-targeted metagenomics further identified the genetic signatures of *Microbacterium*, particularly associated with MT2 and MT3. We earlier revealed unique antimicrobial-resistant and virulent phenotypes in the ISS isolate *M. mcarthurae* (Hill et al., 2025). Similarly, we also showed earlier strong genomic relatedness of ISS strain *M. meiriae* with *M. paraoxydans*, a species found associated with bacteraemia and leukemia (Karouia et al., 2025; Laffineur et al., 2003; Amano et al., 2019). Our present data suggested the succession and persistence of MAO-bearing Actinomycetota at ISS.

A potential osmolarity-related confounding effect may influence chemotaxis assay, as the addition of DA, NH₄OH, or other bacterial metabolites may alter the osmolarity of the assay medium and *C. elegans* is known to exhibit strong osmotaxis. A recent study tested a saturating dose of NH_4_^+^ (2.5 M) on the chemotaxis of *C. elegans* (Marogi et al., 2024), whereas we used 0–50 μM NH_4_^+^ against *C. elegnas.* NGM has a baseline osmolarity of around 50–150 mOsm, which represents “normosmic” or isotonic conditions for *C. elegans* (Medrano et al., 2025). Minimal (15 μL) amounts of test samples (CB, CBD, *E. coli* OP50 control, or SUP) were drop-coated onto NGM agar for the chemotaxis assay, while taking possible confounding effects of osmolality into account. Experiments incorporating osmolarity-matched controls may be necessary ahead to exclude confounding osmotic effects on chemotactic responses if higher doses of NH_4_^+^being tested.

Monoamine oxidases and related amine-oxidizing enzymes are known to act on other biogenic amines such as tyramine, tryptamine, and phenylethylamine, with substrate preference varying among enzymes and organisms (Obata et al., 2022; Barroso and Rodriguez, 1996; Sullivan et al., 1986; Benedetti et al., 1983). Although the observed DA metabolism in *L. virtsii* is mainly attributed to MAO, its DA-specificity versus general amine-oxidase activity remains to be explored. Similarly, the conditions that trigger host neurochemical metabolism in DA-metabolizing phenotypes need further investigation.

## 5 Conclusion

The *in silico* and *in vitro* data support MAO-based DA metabolism in *L. virtsii* and identify modulatory impacts of DA-driven NH_4_^+^ formation on the behaviour of *C. elegans in vivo*. However, possible physiological impacts of other co-existing dopamine catabolites on the eukaryotic model remain unresolved. The comparative genomics and phylogenetic analysis of MAO homologs suggested that DA degradation detected in *L. virtsii* is not an ISS-specific trait and rather represents an ancestral phenotype widespread in occurrence on Earth. The temporal enrichment of MAO-bearing Actinomycetota reflects potential succession and persistence of the present taxa aboard ISS. Further recombinant MAO-based assays, genome-wide expression analyses, and/or knockout-assays may shed more light on MAO and/or alternative deaminases involved in DA degradation and associated chemosensory interaction with model eukaryotes.

## Data Availability

The datasets presented in this study can be found in online repositories. The names of the repository/repositories and accession number(s) can be found in the article/Supplementary material.

## Glossary

ALDH: Aldehyde dehydrogenase
BLAST: Basic Local Alignment Search Tool
CB: Citrate buffer (pH 5.6)
CBD: Citrate buffer supplemented with 1 mM dopamine
cDNA: Complementary DNA
CFU: Colony-forming unit
COMT: Catechol-O-methyltransferase
*cslA*: cellulose synthase-like protein
DA: Dopamine
DOPAL: 3,4-Dihydroxyphenylacetaldehyde
F6_8S_P_1A^T^: Type strain of *Leifsonia virtsii*
F6_8S_P_1B^T^: Type strain of *Leifsonia williamsii*
*gyrB*: DNA gyrase B
HVA: Homovanillic acid
ISS: International Space Station
MAO: Monoamine oxidase
NCBI: National Centre for Biotechnology Information
NGM: Nematode Growth Medium
NH_4_^+^: Ammonium ion
NH_4_OH: Ammonium hydroxide
OrthoANI: Orthologous Average Nucleotide Identity
OD: Optical density
PBS: Phosphate buffered saline
qRT-PCR: Real-time quantitative reverse transcription PCR
*rpoD*: RNA polymerase sigma factor
RT-PCR: Reverse transcription PCR
SUP: Secretome of *L. virtsii* F6_8S_P_1A^T^ grown in CBD
TSA: Tryptic soy agar
TSB: Tryptic soy broth
*tuf*: elongation factor Tu
UV: Ultraviolet
