# Supplementary material for: Temporal enrichment of bacterial monoamine oxidase gene aboard the international space station and neuromodulator impacts of dopamine-driven ammonium formed by space isolate Leifsonia virtsii on Caenorhabditis elegans
Source: Front Microbiol. 2026 Oct 23;17:1943403. doi: 10.3389/fmicb.2026.1943403 (PMC13761221; doi:10.3389/fmicb.2026.1943403)
Supplement: Supplementary file 1 [file Data_Sheet_1.PDF]

Temporal enrichment of bacterial monoamine oxidase gene aboard the International Space Station and neuromodulator impacts of dopamine-driven ammonium formed by space isolate *Leifsonia virtsii* on *Caenorhabditis elegans*

Naikap Mayuri[1], Honagodu Ravichandra Dhanyashree[1,2], Rajesh Padumane Shastry[1,2,3]*, Kasthuri Venkateswaran[4, 5], Georgios Miliotis[6], Nitin Kumar Singh[4,7], Paul Stothard[8], Ananthapadmanabha Bhagwath Arun[1,9], Asif Hameed[1,2,3]*

[1]Division of Microbiology and Biotechnology, Yenepoya Research Centre, Yenepoya (Deemed to be University), Deralakatte, Mangalore-575018, India

[2]*Caenorhabditis elegans* Laboratory Facility (CeLF), Yenepoya Research Centre, Yenepoya (Deemed to be University), Deralakatte, Mangalore-575018, India

[3]Anaerobic & Translational Genomics Centre (ATGC), Yenepoya Research Centre, Yenepoya (Deemed to be University), Deralakatte, Mangalore-575018, India

[4]NASA Jet Propulsion Laboratory (JPL), California Institute of Technology, Pasadena, California, USA

[5]Department of Space Studies, University of North Dakota, Grand Forks, North Dakota, USA

[6]Antimicrobial Resistance and Microbial Ecology Group, School of Medicine, University of Galway, Galway, Ireland

[7]Department of Industrial Relations, Division of Occupational Safety and Health, Oakland, USA

[8]Department of Agricultural, Food and Nutritional Science, University of Alberta, Edmonton, Alberta T6G 2P5, Canada.

[9]Yenepoya International School, Al Khobar, Al Azziziya, Al Amwaj District, Kingdom of Saudi Arabia

ORCID ID

NM: https://orcid.org/0009-0004-0972-5910 mayurinaikap16@gmail.com
DHR: https://orcid.org/0009-0005-3115-8016 dhanyabhat1999@gmail.com
RPS: https://orcid.org/0000-0001-8627-9759 rpshastry@yenepoya.edu.in
KV: https://orcid.org/0000-0002-6742-0873 kjvenkat1955@gmail.com
GM: https://orcid.org/0000-0002-0944-2206 georgios.miliotis@universityofgalway.ie
NKS: https://orcid.org/0000-0001-5344-1190 mail2nitinks@gmail.com
PS: https://orcid.org/0000-0003-4263-969X stothard@ualberta.ca
ABA: https://orcid.org/0000-0003-3140-9266 arunbhagwath@yenepoya.edu.in
AH: https://orcid.org/0000-0003-4445-7080 asifhameed@yenepoya.edu.in

***Correspondence**

Dr. Rajesh P. Shastry, E-mail: rpshastry@yenepoya.edu.in
Dr. Asif Hameed, E-mail: asifhameed@yenepoya.edu.in

Table S1. Details of validly established strains of *Leifsonia* analysed in this study

Sl. No.	Species name & Author	GenBank Accession	Genome size (Mb)	Origin	Date of genome deposition	MAO	COMT	ALDH
1	*L. virtsii* Simpson et al. 2024	GCA_030433675.1	4.2	Crew quarters, International Space Station	Jul 11, 2023	1	1	1
2	*L. williamsii* Simpson et al. 2024	GCA_030433685.1	3.9	Crew quarters, International Space Station	Jul 11, 2023	0	1	1
3	*L. shinshuensis* Suzuki et al. 2000	GCA_013410375.1	4.4	Soil sample from Nagano Prefecture, Japan	Jul 13, 2020	1	1	1
4	*L. soli* Madhaiyan et al. 2010	GCA_013408745.1	3.9	Teak tree rhizosphere soil	Jul 13, 2020	1	1	1
5	*L. cynodontis* (Davis et al. 1984) Suzuki et al. 2000	GCA_000470775.1	2.7	Bermuda grass	Sep 24, 2013	0	1	1
6	*L. lichenia* An et al. 2010	GCA_054780215.1	4.1	Lichen in Japan	Apr 29, 2025	1	1	1
7	*L. xyli* (Davis et al. 1984) Evtushenko et al. 2000	GCA_001647635.1	2.7	Xylem of the wood	May 19, 2016	1	1	1
8	*L. aquatica* (ex Leifson 1962) Evtushenko et al. 2000	GCA_014190775.1	4.4	Nematode galls on Poa annua	Aug 14, 2020	1	1	1
9	*L. naganoensis* Suzuki et al. 2000	GCA_013410615.1	4.3	Soil sample from Nagano Prefecture, Japan	Jul 13, 2020	1	1	1
10	*L. poae* Evtushenko et al. 2000	GCA_027921845.1	4.1	Nematode galls on *Poa annua*	Jan 19, 2023	1	1	1
11	*L. bigeumensis* Dastager et al. 2008	GCA_039537265.1	2.8	Soil sample from Bigeum Island, Korea	Feb 23, 2024	0	1	1
12	*L. stereocauli* Zuo et al. 2025	GCA_038432415.1	3.6	Stereocaulon lichen	Apr 23, 2024	0	1	1
13	*L. tongyongensis* (Kim et al. 2014) Gtari et al. 2025	GCA_010686705.1	4.1	Tongyong region in the Republic of Korea	Feb 15, 2020	0	1	1
14	*Leifsonia antarctica* Pindi et al. 2009	NA	NA	Marine sediment sample, Antarctica	NA	NA	NA	NA

Abbreviation: MAO, monoamine oxidase; COMT, catechol-O-methyltransferase; ALDH, aldehyde dehydrogenase; 1, present; 0, absent; NA, not available.

Table S2. Details of reclassified *Leifsonia* and detection of MAO

Basonym	Reclassified taxon and author
L. aurea	*Rhodoglobus aureus* (Reddy et al. 2003) An et al. 2011
L. flava	*Orlajensenia leifsoniae* Gtari et al. 2025
L. ginsengi†	*Herbiconiux ginsengi* (Qiu et al. 2007) Behrendt et al. 2011
L. kafniensis†	*Glaciibacter kafniensis* (Pindi et al. 2009) Gtari et al. 2025
L. kribbensis†	*Pseudolysinimonas kribbensis* (Dastager et al. 2009) Heo et al. 2019
L. pindariensis†	*Microterricola pindariensis* (Reddy et al. 2008) Dhotre et al. 2017
L. psychrotolerans†	*Glaciibacter psychrotolerans* (Ganzert et al. 2011) Gtari et al. 2025
L. rubra	*Salinibacterium rubrum* (Reddy et al. 2003) Gtari et al. 2025

† MAO positive

Table S3. Details of the primers used for gene expression analysis of *Leifsonia virtsii*

Sl. No.	Gene/Enzyme	PRIMERS	Primer sequence (5' -3')	Length (bp)	Tm (°C)	GC (mol%)	Amplicon size (bp)
1	Monoamine oxidase	MAO_F	TGGTGGTCAGCTTCTTCGTC	20	59.97	55	386
		MAO_R	ATTCGAAATCAGCCACACGC		59.55	55	
2	16S rRNA gene	16S_F	CATTCGGCATCGATCCGTCC	20	61.28	60	279
		16S_R	CCTGGGTGAGCGAGATGAAT		59.53	55	
3	DNA gyrase B	gyrB_F	TCGGAGCACGAGTACGTCTA	20	60.11	55	306
		gyrB_R	CAGATGTCGAGGAACCGGAC		60.18	60	
4	elongation factor Tu	tuf_F	GGTCACGCCGACTACATCAA	20	60.11	55	333
		tuf_R	GTCCATGAGGTCCAGAACCG		60.11	60	
5	RNA polymerase sigma factor	rpoD_F	AGACGACATCCCCGTCTACT	20	59.74	55	316
		rpoD_R	GTCAAGGAACTGCATGCCAC		59.76	55	
6	cellulose synthase-like protein	cslA_F	GGTCAAGACGACCGCTTACA	20	60.04	55	375
		cslA_R	TACCACTTGGCGACGTACAC		60.04	55	

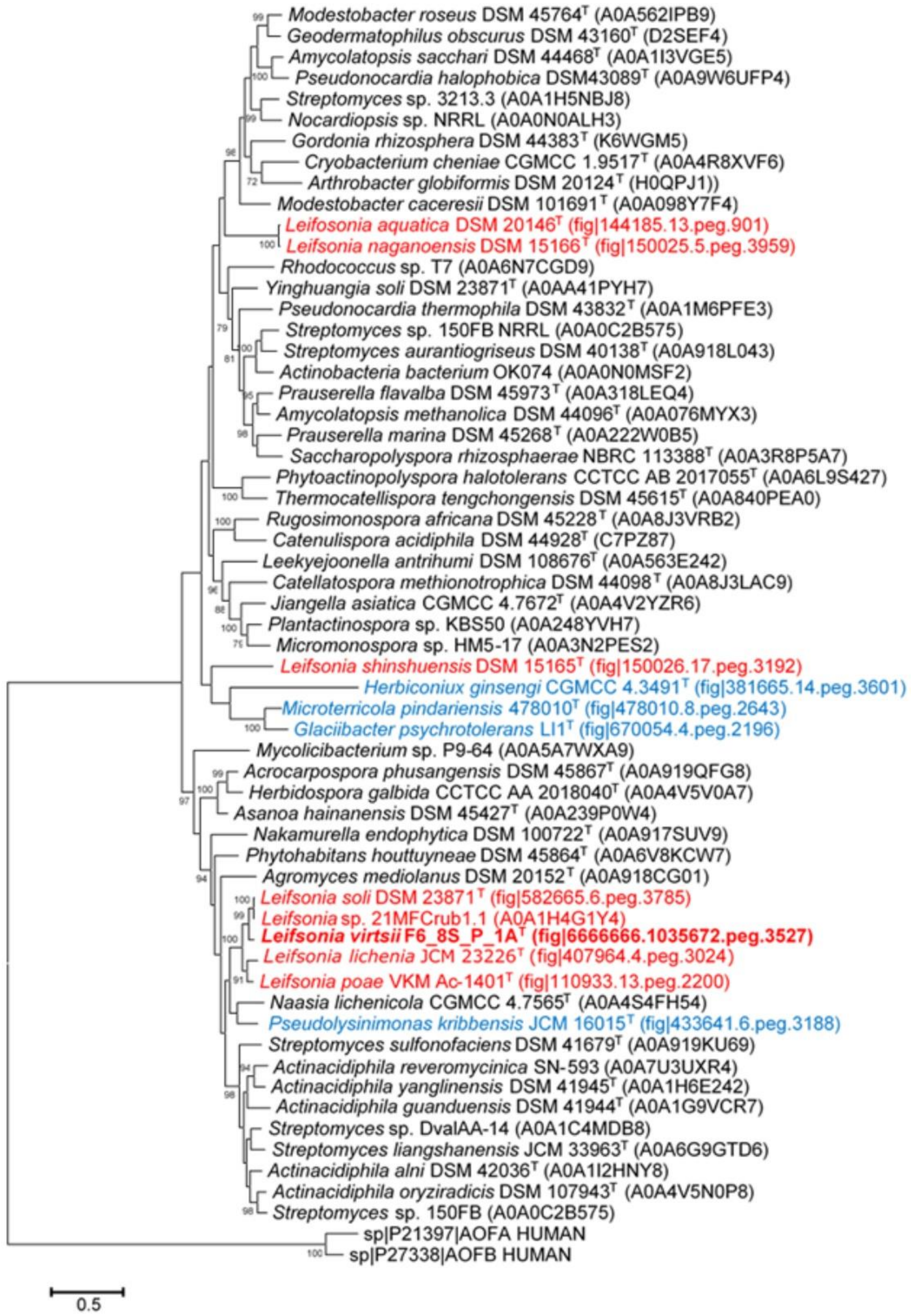

Fig. S1. Maximum likelihood phylogeny of amino acid sequences corresponding to monoamine oxidases (MAO), showing the relationship between *Leifsonia virtsii* F6_8S_P_1A^T (red bold font) and other related isolates originating from terrestrial or aquatic habitats. Taxa given in red font are authentic type strains of *Leifsonia*. Taxa shown in blue font are *Leifsonia* reclassified to other taxa. NCBI/UniProt accession numbers or RAST locus tags for individual sequences are given in respective parenthesis. Bootstrap values of >70% after 1000 replications are shown at the branching nodes. Scale bar: 0.5 substitutions per site. MAO sequences of humans were used as an outgroup.

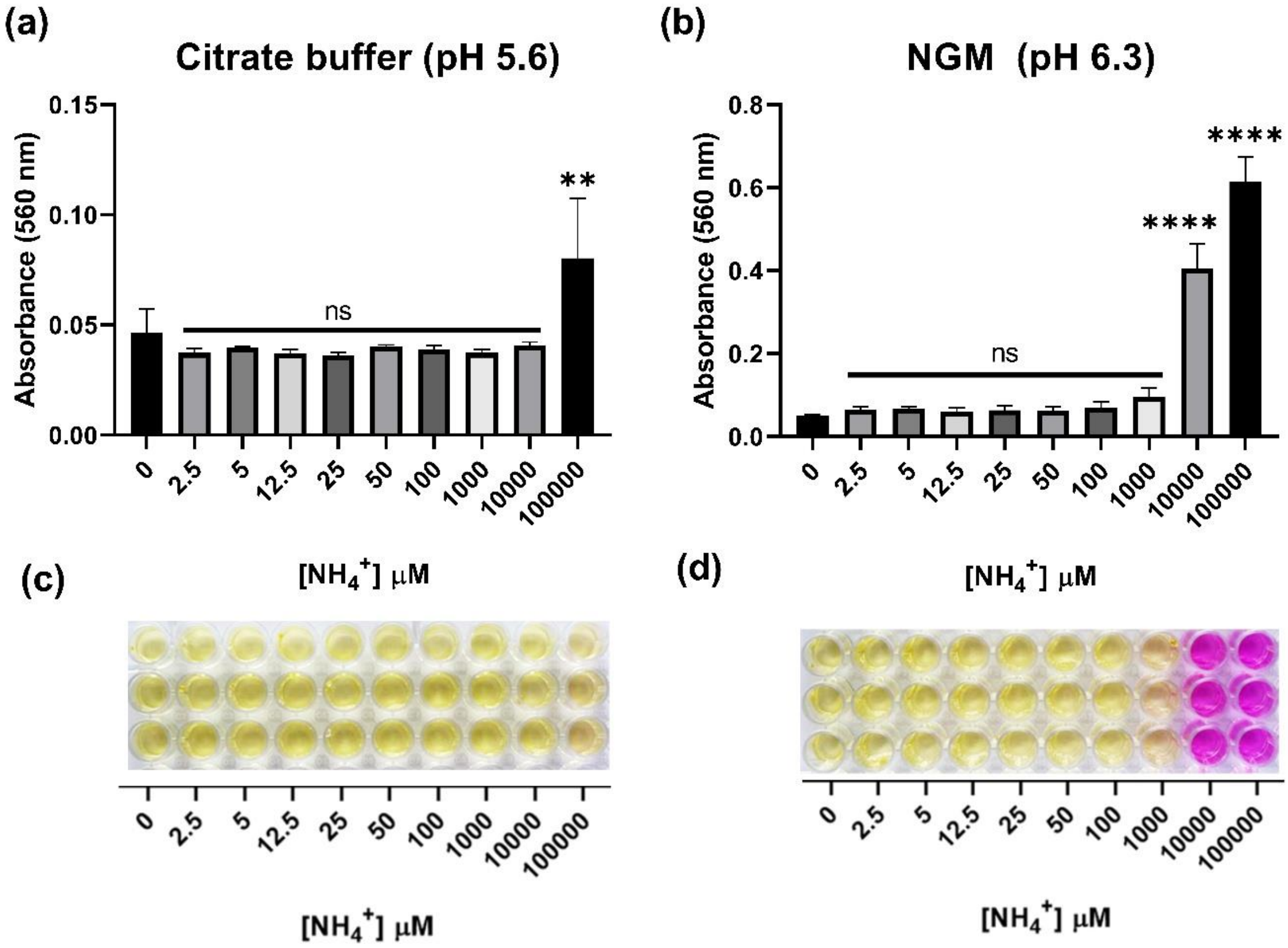

Fig. S2. The impact of various concentrations of ammonium on the pH of citrate buffer (pH 5.6) (CB) and nematode growth medium (pH 6.3) (NGM) under abiotic conditions. Phenol red was used as an indicator and readings were taken at 560 nm. Statistical comparisons were made using one-way ANOVA followed by Tukey's multiple comparisons test. Error bar, mean (n = 3) ± SD and *$P < 0.1$, **$P < 0.05$, ***$P < 0.01$, ****$P < 0.0001$; ns, non-significant.

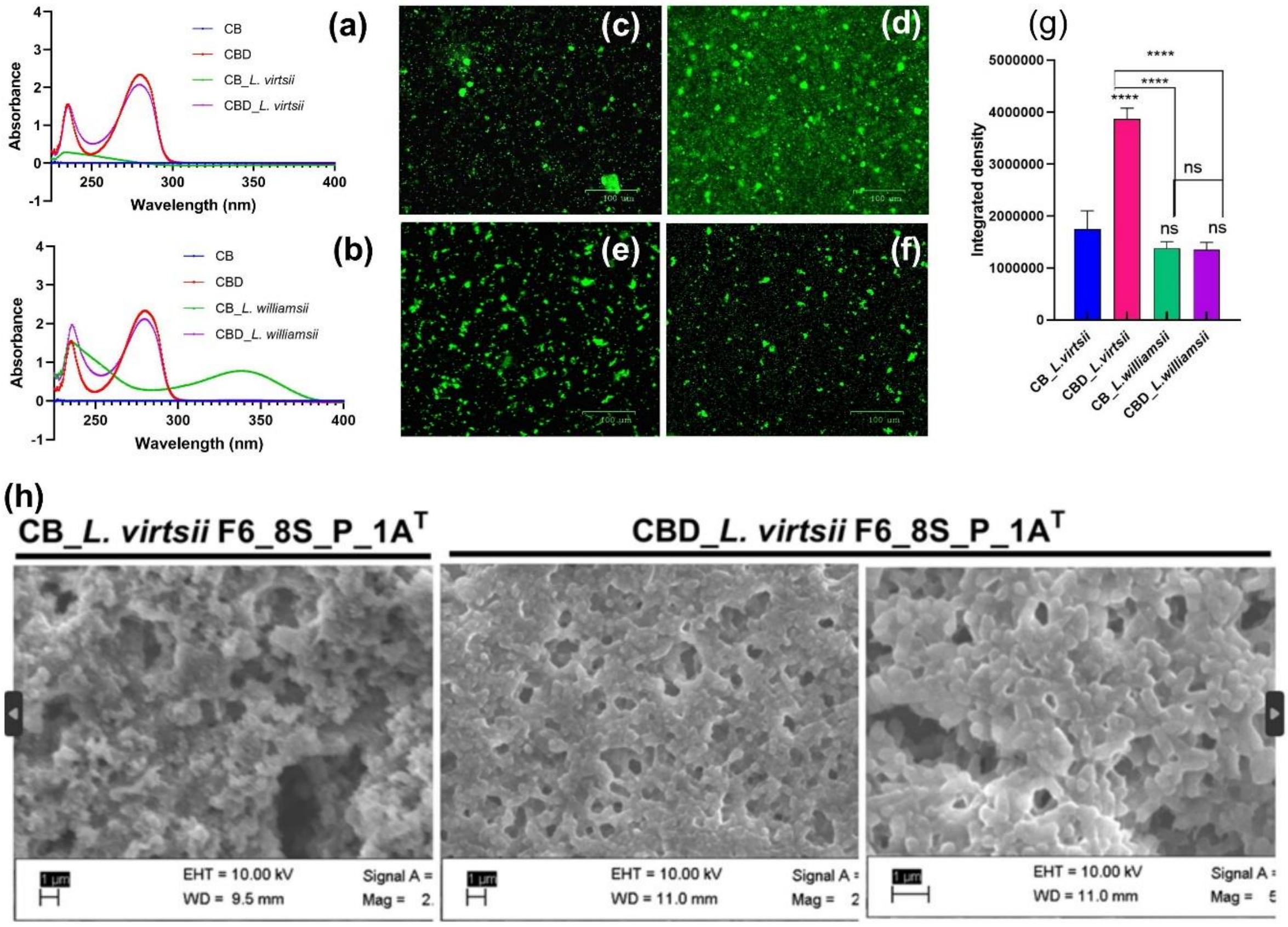

Fig. S3. Spectrophotometric and microscopic evidence for dopamine (DA)-driven biofilm formation in *Leifsonia virtsii* F6_8S_P_1A^T. UV–visible spectra obtained for citrate buffer (pH 5.6) (CB) culture media with and without DA (CB and CBD, respectively) supplementation, inoculated with *L. virtsii* (a) and *L. williamsii* (b); cell-free CB and CBD blanks are shown. Fluorescence images taken for *L. virtsii* in the absence (c) and presence of DA (d). Fluorescence images taken for *L. williamsii* in the absence (e) and presence of DA (f); scale bar, 100 μm. The fluorescence measurement intensity obtained for *L, virtsii* and *L. williamsii* when grown in CB and CBD are shown. Statistical comparisons were made using one-way ANOVA followed by Tukey's multiple comparisons test. Error bar, mean (n = 8) ± SD and $*P < 0.1$, $**P < 0.05$, $***P < 0.01$, $****P < 0.0001$; ns, non-significant. Scanning electron micrographs obtained for *L. virtsii* grown in CB (h) and CBD (i–j). Scale bar, 1 μm.

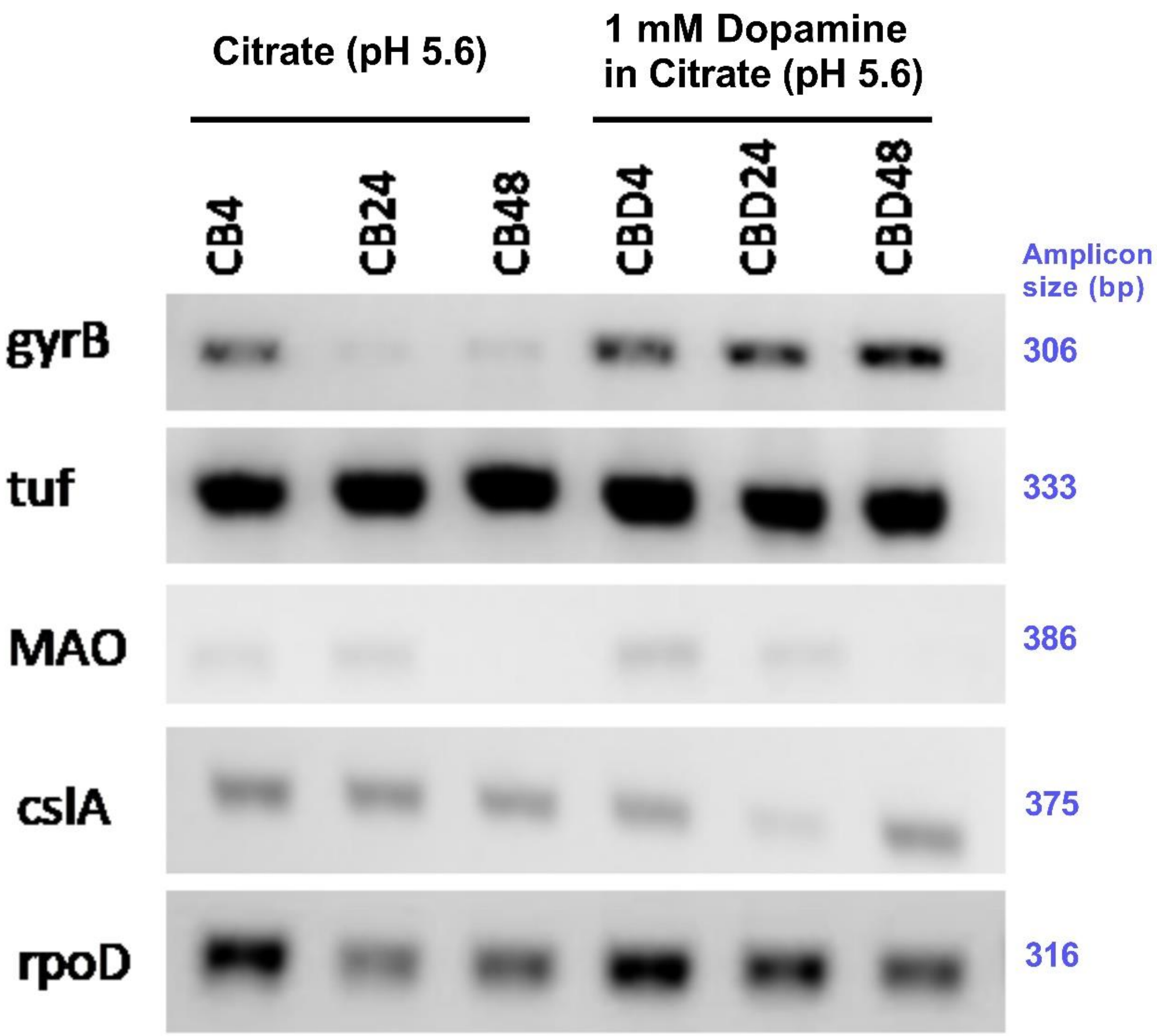

Fig. S4. Reverse transcription PCR (RT–PCR) analysis of the time course of gene expression in *Leifsonia virtsii* F6_8S_P_1A^T in citrate buffer (pH 5.6) in the presence and absence of 1 mM dopamine (DA). Trends in the expression of genes encoding DNA gyrase B (*gyrB*), elongation factor Tu (*tuf*), monoamine oxidase (*MAO*), and cellulose synthase-like protein (*cslA*), respectively, were used as markers for DNA replication and transcription, translation, DA catabolism/NH_4^+ formation and biofilm formation. A housekeeping gene encoding RNA polymerase sigma factor (*rpoD*) was used as a reference.

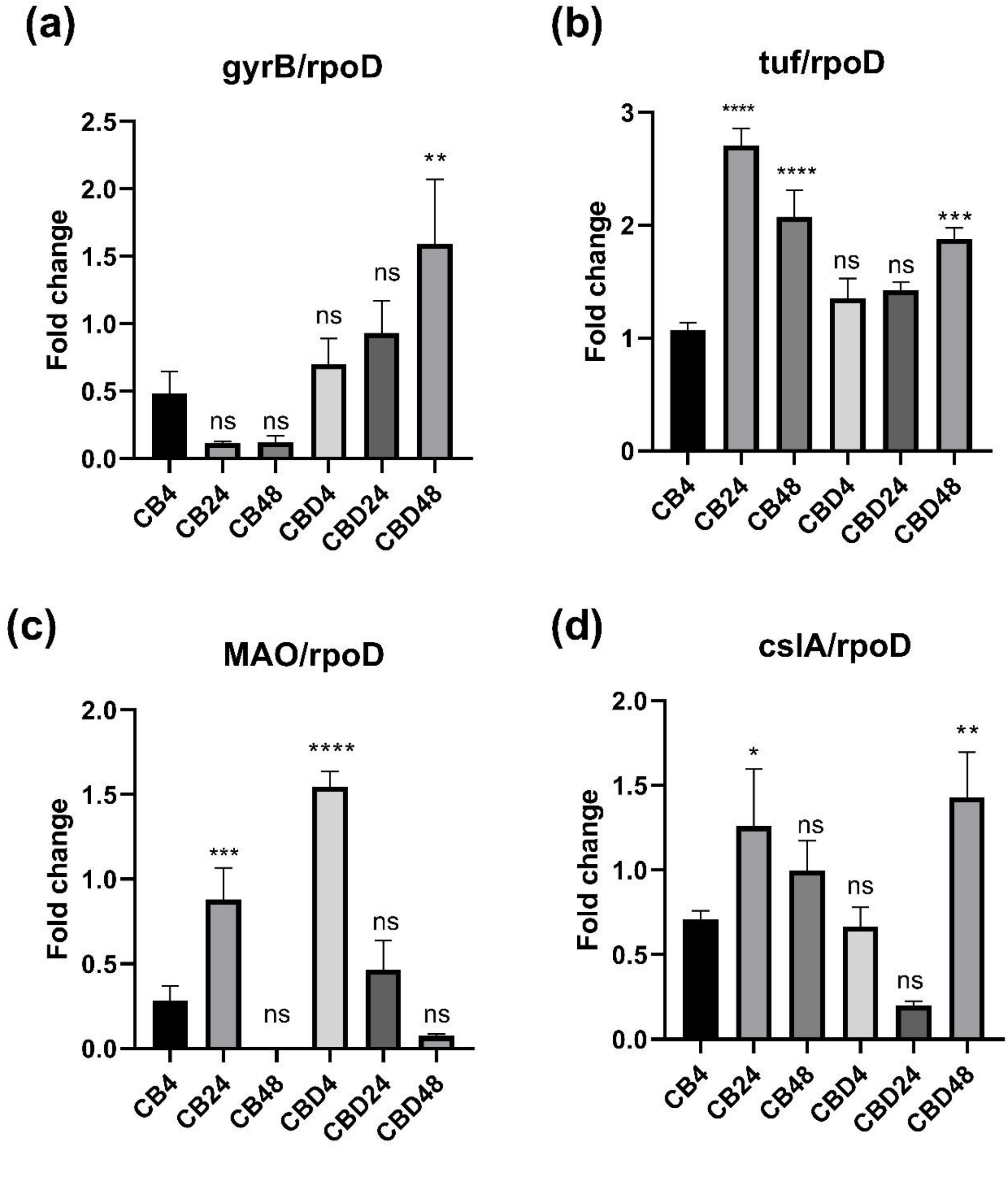

Fig. S5. Densitometry for reverse transcription PCR (RT–PCR) amplicons. The time course of gene expression in *Leifsonia virtsii* F6_8S_P_1A^{T} in citrate buffer (pH 5.6) (CB) in the presence and absence of 1 mM dopamine (DA), as determined through the ImageJ tool. Trends in the expression of genes encoding DNA gyrase B (*gyrB*), elongation factor Tu (*tuf*), monoamine oxidase (*MAO*), and cellulose synthase-like protein (*cslA*), were used as markers for DNA replication and transcription, translation, DA catabolism/NH_4^+ formation and biofilm formation, respectively. A housekeeping gene encoding RNA polymerase sigma factor (*rpoD*) was used as a reference. CB4, CB24 and CB48 represent RNA samples retrieved from citrate buffer (pH 5.6) (CB) at 4, 24 and 48 h; CBD4, CBD24 and CBD48 represent RNA samples retrieved from1 mM DA-supplemented CB at 4, 24 and 48 h. Statistical comparisons were made using one-way ANOVA followed by Tukey's multiple comparisons test. Error bar, mean (n = 3) ± SD and $*P < 0.1$, $**P < 0.05$, $***P < 0.01$, $****P < 0.0001$; ns, non-significant.
